# Electrospun Gelatin Scaffolds with Incorporated Antibiotics for Skin Wound Healing

**DOI:** 10.3390/ph17070851

**Published:** 2024-06-28

**Authors:** Katarina Virijević, Marko Živanović, Jelena Pavić, Luka Dragačević, Biljana Ljujić, Marina Miletić Kovačević, Miloš Papić, Suzana Živanović, Strahinja Milenković, Ivana Radojević, Nenad Filipović

**Affiliations:** 1 Institute for Information Technologies, University of Kragujevac, 34000 Kragujevac, Serbia; marko.zivanovic@uni.kg.ac.rs (M.Ž.); jelena.pavic@uni.kg.ac.rs (J.P.); 2 Institute of Virology, Vaccines and Sera “Torlak”, 11000 Belgrade, Serbia; ldragacevic@torlak.rs; 3 Department of Genetics, Faculty of Medical Sciences, University of Kragujevac, 34000 Kragujevac, Serbia; biljana.ljujic@fmn.kg.ac.rs; 4 Department of Histology and Embryology, Faculty of Medical Sciences, University of Kragujevac, 34000 Kragujevac, Serbia; marinamk@medf.kg.ac.rs; 5 Department of Dentistry, Faculty of Medical Sciences, University of Kragujevac, 34000 Kragujevac, Serbia; papic@fmn.kg.ac.rs (M.P.); stomatologija@medf.kg.ac.rs (S.Ž.); 6 Faculty of Engineering, University of Kragujevac, 34000 Kragujevac, Serbia; strahinja.milenkovic@fink.rs (S.M.); fica@kg.ac.rs (N.F.); 7 Department of Biology and Ecology, Faculty of Natural Sciences, University of Kragujevac, 34000 Kragujevac, Serbia; ivana.radojevic@pmf.kg.ac.rs; 8 BioIRC—Bioengineering Research and Development Center, 34000 Kragujevac, Serbia

**Keywords:** electrospinning, tissue engineering, gelatin scaffolds, wound healing, antibiotic agents

## Abstract

Recent advances in regenerative medicine provide encouraging strategies to produce artificial skin substitutes. Gelatin scaffolds are successfully used as wound-dressing materials due to their superior properties, such as biocompatibility and the ability to mimic the extracellular matrix of the surrounding environment. In this study, five gelatin combination solutions were prepared and successfully electrospun using an electrospinning technique. After careful screening, the optimal concentration of the most promising combination was selected for further investigation. The obtained scaffolds were crosslinked with 25% glutaraldehyde vapor and characterized by scanning electron microscopy, energy-dispersive X-ray spectroscopy, and Fourier-transform infrared spectroscopy. The incorporation of antibiotic agents such as ciprofloxacin hydrochloride and gentamicin sulfate into gelatin membranes improved the already existing antibacterial properties of antibiotic-free gelatin scaffolds against *Pseudomonas aeruginosa* and *Staphylococcus aureus*. Also, the outcomes from the in vivo model study revealed that skin regeneration was significantly accelerated with gelatin/ciprofloxacin scaffold treatment. Moreover, the gelatin nanofibers were found to strongly promote the neoangiogenic process in the in vivo chick embryo chorioallantoic membrane assay. Finally, the combination of gelatin’s extracellular matrix and antibacterial agents in the scaffold suggests its potential for effective wound-healing treatments, emphasizing the importance of gelatin scaffolds in tissue engineering.

## 1. Introduction

The skin is the largest organ of the human body. It is responsible for several important functions in the areas of perception, thermoregulation, and the immune system, as well as providing physical protection against various external pathogens [1]. The formation of wounds, chronic inflammation, and prolonged complications due to injuries, still represent a challenge for modern surgery. The first use of dressing material dates to 1880, when Joseph Gamgee used a sandwiched dressing of cotton and wool to treat a skin wound [2]. In comparison to some traditional wound-dressing materials, discovering new innovative materials to replace the old known ones, such as gauzes and bandages, is becoming closer to reality due to the superior capabilities of newly designed artificial materials.

The development of tissue engineering as a branch of modern biomedicine provides new opportunities to solve various health conditions. Artificial nanomaterials are one of the essential types of medical devices, especially those obtained by versatile electrospinning technology [3]. These kinds of materials possess advanced properties, providing the possibility of covering, protecting, and creating an appropriate microenvironment by imitating the structure of the extracellular matrix (ECM) of injured organs/tissue [4].

Electrospinning is an efficient process that can be used to produce continuous nanofibers with an average diameter of tens to hundreds of nanometers [5]. The structure of fibers with such a diameter range allows the creation of a porous structure that is ideal for tissue engineering applications. In electrospinning, the process parameters have a strong influence on the final structure and geometry of the nanofibers [6]. Therefore, the optimization of process parameters such as solution flow rate, applied voltage, temperature, and humidity of the environment, as well as solution properties, is key to a successful nanofiber structure [7].

The main components in electrospinning technology for obtaining artificial materials are based on different types of polymers and natural polymers. Consequently, natural polymers have attracted much attention in the field of skin tissue engineering and drug delivery due to their properties such as biocompatibility, biodegradability, non-toxicity, and low cost [8]. One of the most common representative natural polymers is gelatin. Gelatin is a natural polymer with strong polarity due to its hydrogen bonds, constituting a 3D macromolecular network (double or triple helix) with low mobility [9]. The good performance of the gelatin structure provides a proper environment for skin healing and reconstruction, allowing unhindered cell proliferation, differentiation, and migration within the damaged tissue. Gelatin scaffolds are strongly favorable due to their flexible porosity and biocompatibility for soft-tissue engineering or artificial-skin engineering [10].

Modern approaches include the use of nanomaterials that can facilitate the controlled release of antibiotics. Nanomaterials have been widely used in the last decade for the successful incorporation of drugs and their gradual release. Using these advantages, the electrospinning technique has attracted considerable attention in the development of wound-dressing materials designed to accelerate healing processes while increasing biocompatibility.

Regarding the selection of antibiotics in this study, ciprofloxacin hydrochloride, one of them, is a potent quinolone broad-spectrum antibacterial drug synthesized by Bayer Pharmaceuticals in Germany in 1983 [11]. Ciprofloxacin primarily kills bacteria by inhibiting bacterial DNA gyrase and has potent antibacterial activity against Gram-negative, and Gram-positive organisms. Notably, ciprofloxacin is characterized by its hydrophilic nature, coupled with low toxicity and drug resistance [12].

Another one, gentamicin sulfate is an aminoglycoside that is commonly used to treat and prevent bacterial infections [13]. It is effective in treating wounds when used in combination with other materials, especially on naturally derived nanofiber dressings [14].

However, the applications of electrospun gelatin nanofibers are limited by their chemical composition and behavior in a physiological environment. Gelatin is immediately soluble in water and unsuitable for applications in tissue engineering. As a result, additional treatment, such as crosslinking, is required to modify the intermolecular bonds in the gelatin structure, providing the water stability and appropriate structure for cell adhesion. Based on previous studies, glutaraldehyde (GA) is a widely used crosslinking agent, particularly for gelatinous fibers [15]. The high crosslinking agent efficiency and low cost provide significant hydrolysis resistance and mechanical properties to gelatin nanofibers [16]. In recent years, there has been an increase in interest in using green-friendly crosslinking agents instead of stronger organic ones. This is due to the trend toward using more natural agents with no or low toxic grades. However, GA has a certain dose of toxicity. Still, through its properly followed evaporation from nanofibers after treatment, neutralization has been successfully achieved, which is consistent with the literature data and results represented in this article [17].

In this study, we present a new approach: gelatin scaffolds with incorporated antibiotic agents, produced by the electrospinning technique, designed specifically for wound-healing applications. Five different gelatin solution combinations were designed and tested by varying certain process parameters. One of the most suitable chemical compositions was selected for further investigation. In order to improve their properties, the gelatin scaffolds were subjected to chemical crosslinking with 25% GA vapor. Comprehensive characterization was conducted using high-essential methods, including scanning electron microscopy (SEM), energy dispersive X-ray spectroscopy (EDS), and Fourier-transform infrared spectroscopy (FTIR), along with assessments of water uptake, degradation, cellular response, and microbial activity.

**Figure 1:**
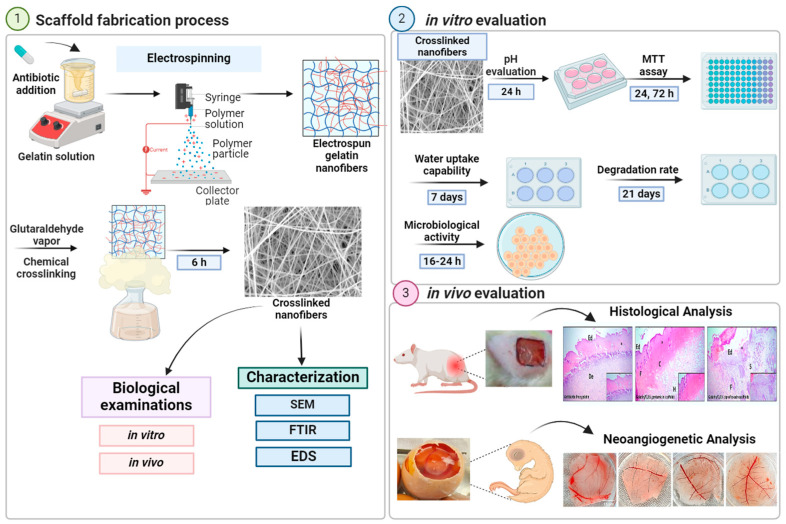
Scaffold fabrication by electrospinning technique followed by in vitro and in vivo evaluations.

In vivo experiments, such as wound healing on the rat animal model and neoangiogenesis assessment on the chicken embryo, are conducted to assess the efficacy of fabricated scaffolds in promoting wound closure and stimulating the formation of new blood vessels. Figure 1 shows the conceptualization of our comprehensive work.

This research represents the extraordinary potential of gelatin electrospun scaffolds for in vivo wound healing, synergistically promoting the process of neoangiogenesis. The findings of this study highlight eminent progress in this area.

## 2. Results

### 2.1. Crosslinking

Gelatin is classified as a hydrophilic polymer because of its abundant hydroxyl groups, which promote strong hydrogen bonding and rapid water solubility. In this study, a successful crosslinking reaction was achieved to address the immediate dissolution of gelatin scaffolds in a physiological environment. The crosslinking was performed after the electrospinning process.

**Scheme 1:**
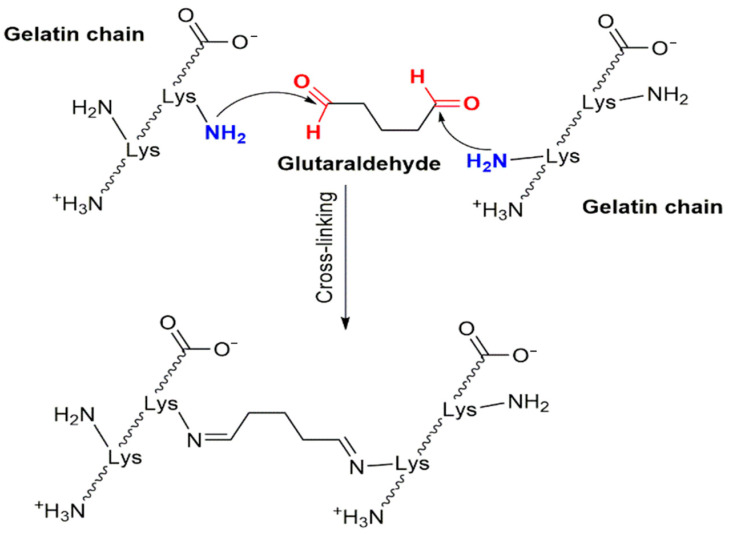
The mechanism of GA–gelatin crosslinking.

**Figure 2:**
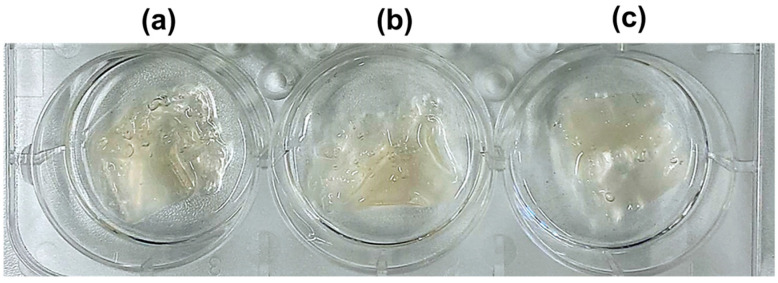
Crosslinked gelatin scaffolds in PBS saline. (**a**) gelatin, (**b**) gelatin/0.1% ciprofloxacin, and (**c**) gelatin/0.1% gentamicin scaffolds.

The stability of crosslinked gelatin nanofibers was evaluated in phosphate buffer saline (PBS) to simulate physiological conditions (37 °C, 5% CO_2_). Initially, the non-crosslinked nanofibers were easily dissolved after immersion in the aqueous solution. However, after the crosslinking process, using GA vapor for 6 h, significant changes were observed. The crosslinked gelatin nanofibers were turned into insoluble scaffolds, indicating the efficiency of the crosslinking process. The obtained nanofibers, presented in Figure 2, showed reduced protein solubility, with improved mechanical stability of the gelatin scaffolds. The chemical reaction of crosslinking between GA and gelatin is represented in Scheme 1.

### 2.2. The Effect of the Acidity Content and pH Value of Nanofibers in a Physiological Environment

The influence of the pH value of a medium affects the overall behavior of cells. The pH of acetic acid-derived gelatin scaffolds has a crucial impact on cell behavior, viability, and tissue regeneration. In tissue engineering applications, adjusting pH to a physiologically relevant range is critical for ensuring biocompatibility and promoting positive cellular responses. Therefore, an assessment of the pH values of nanofibrous scaffolds containing acid content after 24 and 72 h was performed.

Noticeable color changes were observed in the medium after exposure to antibiotic-loaded and antibiotic-free scaffolds. For the control samples (medium without gelatin fibers), the color was pink/purple (Supporting Information, Figure S1), corresponding to a pH of approximately 8.2 based on the phenol red scale. There was no significant difference in pH values between antibiotic-free and antibiotic-loaded scaffolds after 24 and 72 h. Specifically, after 24 h of exposure, both antibiotic-free and antibiotic-loaded scaffolds showed the same pH of 7.7 based on the phenol red scale, indicating an optimal environment for cell growth. Over time, the color of the medium gradually changed from red to a weak orange-pink shade (pH 7.5), as observed in Figure S1. This method provides a preliminary detail on the acidic–basic behavior of the material, which can be useful for toxicity assessment.

### 2.3. Morphology Analyses of Nanofibers

**Figure 3:**
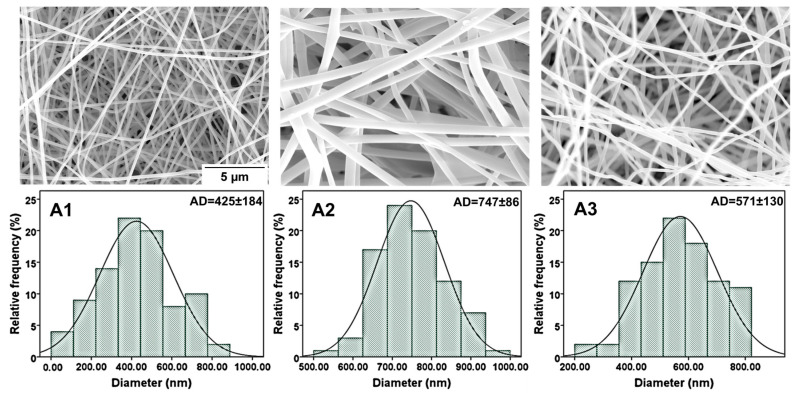
SEM images and their corresponding histograms of the fiber diameters of A1—antibiotic-free gelatin, A2—gelatin/0.1% ciprofloxacin, A3—gelatin/0.1% gentamicin. The scale bar is 5 µm.

The morphology of crosslinked gelatin nanofibers was obtained during the SEM analysis. The SEM images of antibiotic-free gelatin nanofibers and the gelatin nanofibers consisting of 0.1% antibiotic agents are presented in Figure 3. As can be seen, smooth and randomly distributed nanofibers were obtained in all examined samples.

The fiber diameters were measured using ImageJ software package (Version 1.48), and their distribution curves were presented along the SEM micrographs. The average fiber diameters of antibiotic-free gelatin and antibiotic-loaded gelatin nanofibers ranged from 425 to 747 nm. Interestingly, the presence of antibiotics caused structural changes in the nanofibers, resulting in an increased fiber diameter. Specifically, nanofibers containing antibiotics showed larger average diameters compared to their antibiotic-free scaffolds (with an average diameter (AD) of 425 nm for antibiotic-free gelatin and 747 nm and 571 nm for fibers with ciprofloxacin and gentamicin, respectively). There is no significant difference observed in AD between gelatin/0.1% ciprofloxacin and gelatin/0.1% gentamicin nanofibers (747 nm and 571 nm).

#### 2.3.1. FTIR and EDS Analysis

The FTIR spectral analysis in this study was utilized to check the characteristics peaks of non-crosslinked gelatin, crosslinked gelatin, and gelatin with incorporated antibiotics. Firstly, as depicted in Figure S7 (Supplementary Materials), a comparison was made between non-crosslinked and vapor-crosslinked nanofibers [16]. Observing gelatin nanofibers before crosslinking, clear peaks can be detected at 1647 cm^−1^, corresponding to the amide-I group, due to C=O stretching. The peak observed at 1537 cm^−1^ corresponds to the amide-II group because of N–H stretching and C–N bending, and lastly 1243 cm^−1^ relates to the amide-III peak due to C–N stretching and N–H bending vibrations. A sharp peak at 3288 cm^−1^ is a characteristic band of the amide-A group, due to N–H stretching of the amide group. The presence of water is confirmed at 1640 cm^−1^, while 1044 cm^−1^ confirms C–O stretching [18].

**Figure 4:**
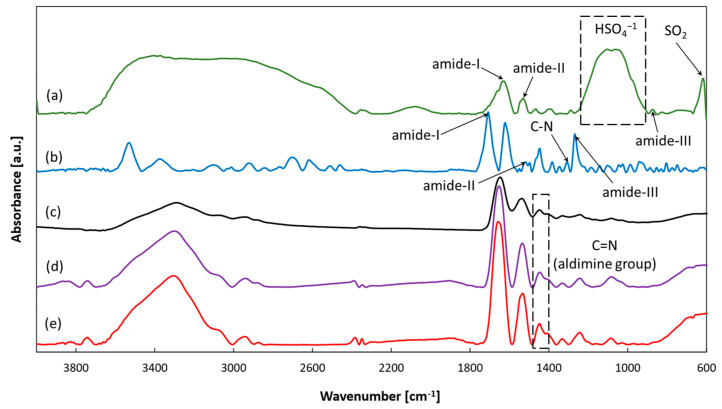
FTIR spectra of (**a**) gentamicin sulfate powder, (**b**) ciprofloxacin hydrochloride powder, (**c**) gelatin nanofibers, (**d**) gelatin/0.1% gentamicin nanofibers, and (**e**) gelatin/0.1% ciprofloxacin nanofibers.

Observing GA-vapor-crosslinked gelatin nanofibers, peaks found at 1653 cm^−1^, 1530 cm^−1^, and 1245 cm^−1^ correspond to amide-I, amide-II, and amide-III, respectively, while the peak at 3283 cm^−1^ represents amide-A. We hypothesize that the peak found at 1450 cm^−1^ depicts crosslinking occurrence due to aldimine linkage (C=N), following the reported literature data [18]. Groups shifting with the addition of GA suggests the existence of intermolecular interaction [19]. Figure 4 describes FTIR spectra of the powder forms of antibiotic agents, antibiotic-free gelatin, and gelatin fibers with incorporated antibiotic agents.

**Figure 5:**
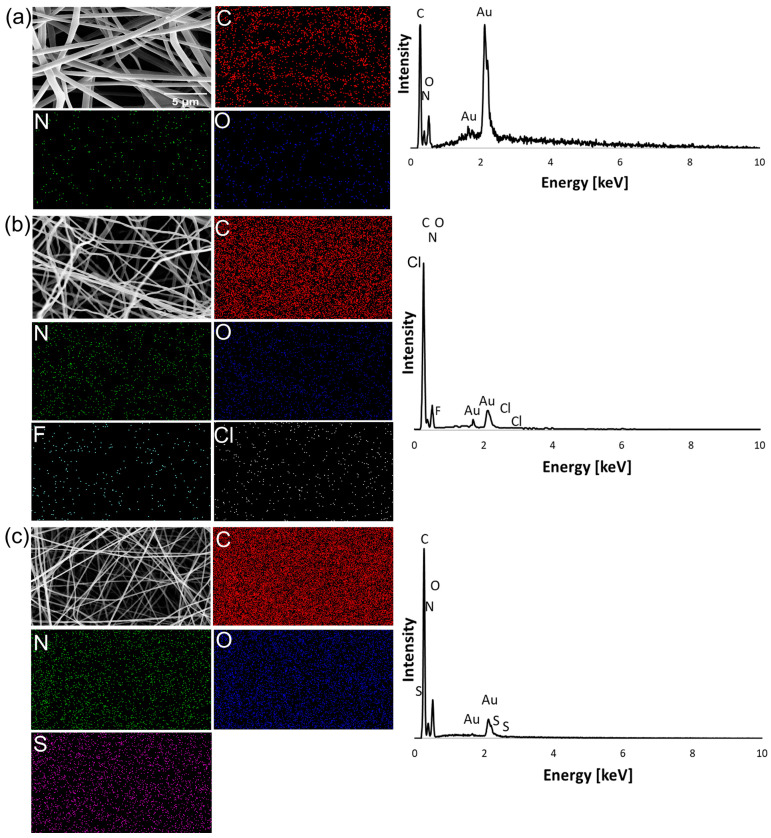
Elemental mapping of (**a**) antibiotic-free gelatin, (**b**) gelatin/0.1% ciprofloxacin, and (**c**) gelatin/0.1% gentamicin nanofibers concerning the distribution of C (red), N (green), O (blue), F (light blue), Cl (white), and S (purple). The scale bar is 5 µm.

The gentamicin powder sample exhibited peaks at the typical absorption bands at 1630, 1533, and 1247 cm^−1^, which can be assigned to the amide-I, amide-II, and amide-III bonds of gentamicin, respectively. The peak observed at 1062 cm^−1^ was due to the HSO_4_^−1^ group, while the peak observed at 617 cm^−1^ was due to the SO_2_ band [20]. In addition, the ciprofloxacin powder sample exhibited peaks at the absorption bands at 1623, 1519, and 1269 cm^−1^, which can be assigned to the amide-I, amide-II, and amide-III bonds of ciprofloxacin, respectively. The peak observed at 1281 cm^−1^ due to C–H stretching is characteristic of ciprofloxacin [21]. Both antibiotic-included gelatin samples showed a 1450 cm^−1^ peak, which confirms crosslinkage. However, no characteristic peaks of gentamicin and ciprofloxacin were observed on the respective spectra, possibly because of their low concentrations, but also characteristic peaks of antibiotics were covered by those peaks of gelatin [22]. To prove that the ciprofloxacin and gentamicin were successfully loaded into the scaffolds, EDS analysis was carried out. Figure 5 shows the EDS mapping of the antibiotic-free gelatin, gelatin/0.1% ciprofloxacin, and gelatin/0.1% gentamicin nanofibers. The chemical structure of ciprofloxacin hydrochloride consists of the fluorine (F) and chlorine (Cl) elements in the structure, while gentamicin sulfate consists of sulfur (S). As shown in Figure 5, F, Cl, and S atoms were present and uniformly distributed, indicating the structural stability of antibiotics and successful attachments to the gelatin nanofibers. These results confirmed strong evidence that both antibiotics were successfully loaded.

#### 2.3.2. Test of Absorption and Degradation

##### Absorption

**Figure 6:**
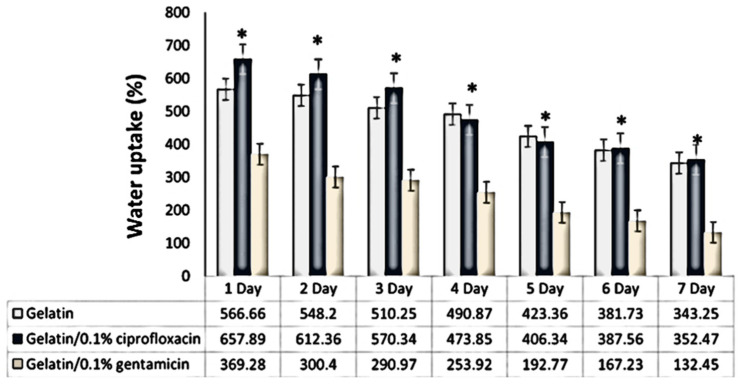
Water uptake of gelatin, gelatin/0.1% ciprofloxacin, and gelatin/0.1% gentamicin nanofibers scaffolds during the 7 days of incubation in PBS (pH = 7.4, 37 °C). Data are shown as mean ± SD on duplicate experiments. * Represents statistically significant from gelatin/0.1% gentamicin at *p* < 0.05.

The degree of absorption, as well as the kinetics of the process, plays an important role in the exchange of gases, liquids, and exudate during the wound-healing process. The water uptake capacity/time diagram of antibiotic-free and antibiotic-loaded scaffolds is presented in Figure 6. PBS was chosen as the solvent to evaluate water uptake by the nanofibrous scaffolds.

The obtained results show the variations in water absorption capacity depending on the type of gelatin sample. The initial rapid increase of water uptake on the first days was followed by a slow, adequate down-rate over time. The degree of water absorption is high during the first days, and this is especially noticeable in the case of the gelatin/0.1% ciprofloxacin and antibiotic-free gelatin nanofibers. The maximum water uptake after 1 day was 566.66% for gelatin, 657.89% for gelatin/0.1% ciprofloxacin, and 369.28% for gelatin/0.1% gentamicin. Similarly, the antibiotic-free gelatin scaffolds showed a high rate of water absorption due to the presence of strong polar amines and carboxyl groups. After the last day, the capacity of water uptake of antibiotic-free gelatin, gelatin/0.1% ciprofloxacin, and gelatin/0.1% gentamicin was 343.25%, 352.47%, and 132.45%, respectively. It is noticeable that gelatin/0.1% ciprofloxacin still had the most notable capacity for water uptake in comparison with the antibiotic-free gelatin and gelatin/0.1% gentamicin scaffolds.

Interestingly, the gelatin/0.1% gentamicin showed the lowest water absorption at all time points, almost half that of gelatin/0.1% ciprofloxacin. In comparison with gelatin/0.1% ciprofloxacin, the water uptake capability of gelatin/0.1% gentamicin was almost twice as low (Figure 6). It is suggested that the presence of gentamicin in the scaffold inhibits water uptake compared to gelatin/0.1% ciprofloxacin and antibiotic-free gelatin scaffolds.

##### Degradation

**Figure 7:**
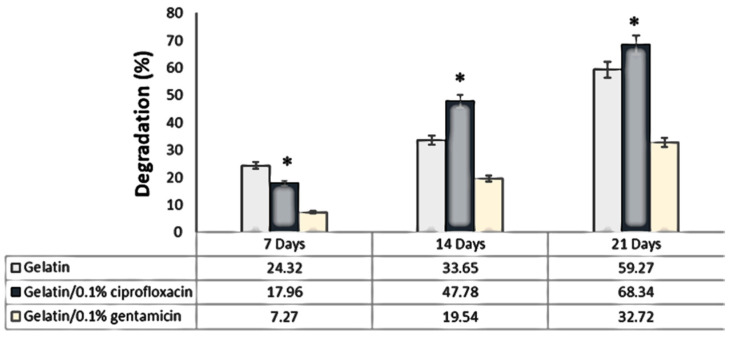
The degradation profile of gelatin, gelatin/0.1% ciprofloxacin, and gelatin/0.1% gentamicin nanofiber scaffolds during the 21 days of incubation in PBS (pH = 7.4, 37 °C). Data are shown as mean ± SD on duplicate experiments. * Represents statistically significant from gelatin/0.1% gentamicin at *p* < 0.05.

As noticed, the high polarity of gelatin, due to the presence of polar groups, accelerates hydrolysis in the aqueous environment. Figure 7 shows the rate of degradation of nanofibrous scaffolds under physiological conditions at 37 °C over 21 days. After 7 days, there were no significant differences in the percentage of degradation rate between the antibiotic-free gelatin and gelatin/0.1% ciprofloxacin scaffolds, while the lowest rate was observed in the gelatin/0.1% gentamicin sample.

At the end of the experiment, it is noticeable that gelatin/0.1% ciprofloxacin had the highest degradation rate (21st day = 68.43%), followed by antibiotic-free gelatin (21st day = 59.27%), and gelatin/0.1% gentamicin (21st day = 32.72%). This redistribution is logically aligned with the observed degree of water absorption.

#### 2.3.3. In Vitro Cytotoxicity Assay

**Figure 8:**
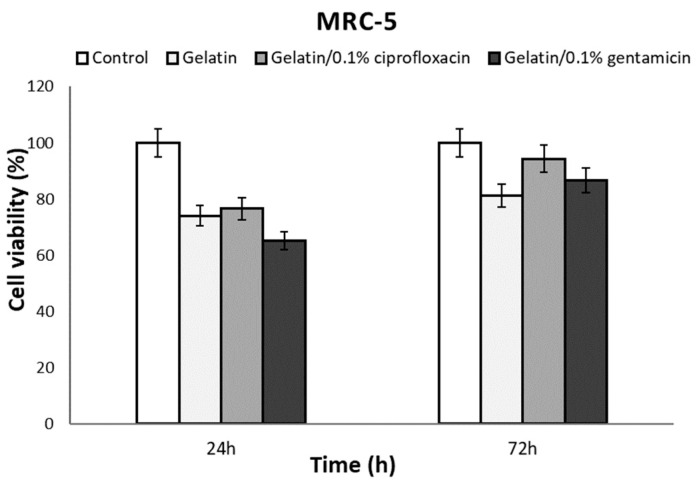
Cytotoxicity test on MRC-5 culture onto antibiotic-free and antibiotic-loaded ciprofloxacin and gentamicin gelatin nanofibers after 24 and 72 h. No significant differences were observed.

The in vitro cytocompatibility of the obtained scaffolds (antibiotic-free gelatin, gelatin/0.1% ciprofloxacin, and gelatin/0.1% gentamicin) was assessed using the MRC-5 cell line after 24 and 72 h of incubation (Figure 8). The results show that all three types of scaffolds are cytocompatible, exhibiting non-toxic effects and promoting increased cell proliferation over time (Figure 8). After 24 h, the percentage of viable cells for antibiotic-free gelatin, gelatin/0.1% ciprofloxacin, and gelatin/0.1% gentamicin was 74.05%, 76.53%, and 65.23%, respectively. Interestingly, after 72 h, we observed an increase in cell proliferation and it was noted at 81.34%, 94.37%, and 86.76%, respectively. The consistent trend was common to each scaffold, and there was no significant difference between the antibiotic-free gelatin and antibiotic-loaded gelatin scaffolds. However, enhanced cell proliferation was observed on the antibiotic-loaded gelatin scaffolds, highlighting the positive influence of the antibiotic compounds on cell growth [22].

Also, as can be seen, the presence of acetic acid and GA did not influence cell growth and proliferation, indicating the successful evaporation of the acetic acid during the electrospinning process and the lack of inhibitory effects of residual GA in the nanofibers on cell activity. Furthermore, GA-crosslinked gelatin nanofibrous scaffolds have been used successfully as microcarriers to promote fibroblast and endothelial cell growth and proliferation [23].

#### 2.3.4. Analysis of the Antibacterial Activity of Gelatin Material Impregnated with Antibiotics

Antimicrobial activity against Gram-positive *Staphylococcus aureus* ATCC 25923 and Gram-negative bacterium *Pseudomonas aeruginosa* ATCC 27853 was evaluated by the disk diffusion method. The results show that the antibiotic-free gelatin scaffold shows a certain inhibition of the growth of the tested bacteria (Table 1). Moreover, considerable antimicrobial activity was observed in the antibiotic-incorporated gelatin scaffolds. The action of the gelatin with antibiotics is the same or similar to the action of the antibiotics themselves in the control. The effects of the same concentrations of antibiotics on filter paper with the tested material (gelatin with antibiotics) in *S. aureus* ATCC 25923 are the same. In the case of *P. aeruginosa* ATCC 27853, a difference is observed, which is with gentamicin in favor of the tested material and with ciprofloxacin in favor of the control (Table 1). The diameter of inhibition of bacterial growth in gelatin with antibiotics is in the range of the action of standard antibiotic discs or significantly prominent in the case of the action of gelatin/0.1% ciprofloxacin against *S. aureus* ATCC 25923 [24].

The micrographic illustration of the antibacterial properties of antibiotic-free and antibiotic-incorporated gelatin scaffolds is presented in the Supplementary Materials.

#### 2.3.5. In Vivo Application

##### Neoangiogenesis Evaluation—CAM Assay Approach

**Figure 9:**
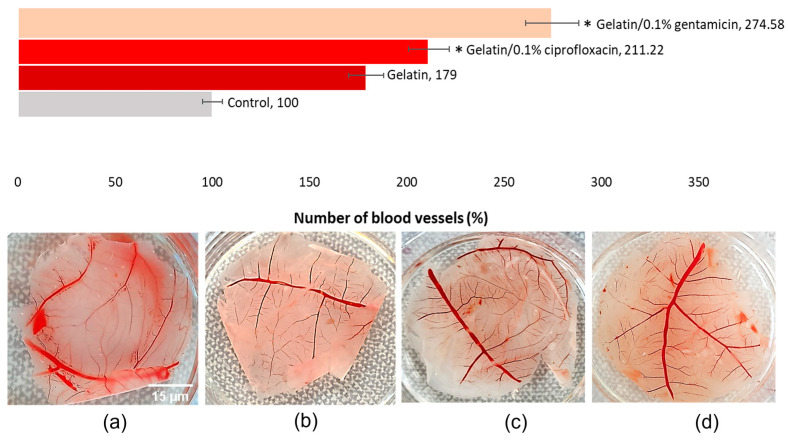
The region under treatment with scaffolds where the percentage of blood vessel area was measured. (**a**) control, (**b**) treatment with gelatin scaffold, (**c**) treatment with gelatin/0.1% ciprofloxacin scaffold, (**d**) treatment with gelatin/0.1% gentamicin scaffold. The scale bar is 15 µm. * Represents statistically significant from control at *p* < 0.05.

The angiogenic potential of biomaterials plays a crucial role in the process of wound healing, promoting blood vessel formation, aiding tissue regeneration, and facilitating wound closure [25]. The impact of the implanted scaffolds on the surface of the egg membrane was evaluated by quantifying the density of blood vessels in the treated region using the chick embryo chorioallantoic membrane (CAM) test. The results reveal that both types of gelatin scaffolds, with and without incorporated antibiotics, did not exhibit toxic effects. Both antibiotic-free and antibiotic-incorporated scaffolds induced increased neoangiogenetic activity in the implantation area, as summarized in Figure 9.

Specifically, treatment with antibiotic-free gelatin scaffolds led to increased formation of blood vessels compared to the control group (179%). Scaffolds containing antibiotic agents, such as gelatin/0.1% ciprofloxacin and gelatin/0.1% gentamicin, have a prominent potential to stimulate the process of formation of new blood vessels (211.22%, and 274.58%, respectively). However, the gelatin/0.1% gentamicin scaffolds showed a more pronounced angiogenic response (274.58%).

#### 2.3.6. In Vivo Wound Healing

To investigate the impact of the electrospun-derived gelatin/0.1% antibiotic-bearing scaffold on wound healing, we carried out an in vivo study. Seven days following the induction of burn wounds, we evaluated the morphological and histological aspects of wound healing in rats. Based on our results, macroscopic evaluation of the wound area revealed enhanced healing due to gelatin/0.1% antibiotic-bearing scaffold treatment compared to the positive control group in rats.

**Figure 10:**
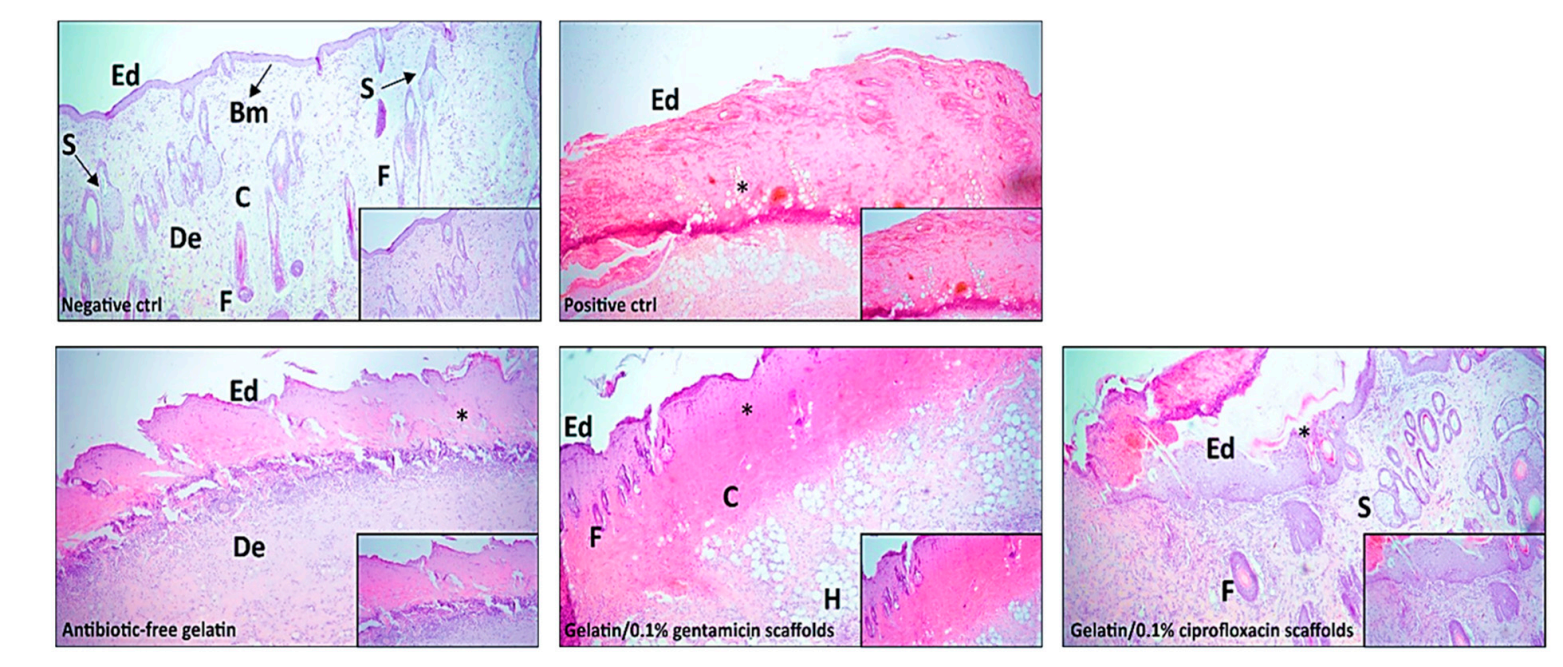
In vivo application of selected scaffolds in the wound-healing process. Representative images of H&E staining of paraffin-embedded sections of negative, positive control after treatment of the scaffolds with incorporated ciprofloxacin and gentamicin (original magnification ×10). Ed: epidermis, Bm: Basement membrane, De: dermis, F: hair follicles, S: sebaceous gland, C: collagen fibers, H: hypodermis, * burn area.

To analyze the histological findings, images of healthy skin (negative control), burned untreated skin (positive control), and treated skin are shown in Figure 10. Healthy skin (negative control) has preserved structures of the epidermis, dermis, and hypodermis, undamaged squamous layered epithelium, and regular distribution of collagen and elastic fibers in the dermis. On the contrary, the sections of burned untreated skin (positive control) displayed necrotic epidermis exfoliated with subsequent separation from the dermis. Areas of coagulative necrosis with neutrophilic infiltration and granulation tissue formation were also noticed in sections of untreated skin (Figure 10).

The obtained histopathological results show notably higher tissue regeneration and repair in all treated groups compared to the positive control group. Also, as expected, the antibiotic-free gelatin group showed an interruption in the resolution of the wound, greater sites of inflammation, and lower deposition of extracellular matrix compared to gelatin/0.1% antibiotic-bearing scaffold groups (Figure 10). The most pronounced healing was achieved in the group with gelatin/0.1% ciprofloxacin scaffolds. Analyzed tissue sections from the group with gelatin/0.1% ciprofloxacin scaffolds demonstrated increased cornification of the stratum corneum, enhanced maturation of the epidermis, reduced numbers of inflammatory cells, and a more orderly arrangement of collagen fibers in the dermis, indicating improved skin regeneration (Figure 10). Also, newly formed blood vessels were determined in a group with gelatin/0.1% ciprofloxacin scaffolds, which achieved neoangiogenesis, ensured adequate blood supply to the injured area, and correspondingly promoted and supported the repair and remodeling of the damaged tissue (Figure 10).

## 3. Discussion

The development of naturally derived antimicrobial wound dressings, such as antibiotic-loaded gelatin nanofibers, addresses a critical need in the field of regenerative medicine. The nanofibrous structures of gelatin scaffolds closely mimic the natural extracellular matrix, providing a favorable microenvironment for cell adhesion, proliferation, and tissue regeneration [26]. The main goal of this study was to fabricate natural scaffolds as biomaterials for regenerative properties. Among these, the ability to incorporate commercially known antibiotics into the gelatin scaffolds enhances their therapeutic use. Recently, the using of natural polymers, such as gelatin, chitosan, alginate, etc., has attracted a lot of attention in the field of tissue engineering [27,28,29]. However, the specific structure of gelatin, in this case, requires stabilization for further biomedical investigations. As noted above in the results of this study, gelatin nanofibers dissolve in physiological conditions. This is explained by the initial chemical structure of gelatin. In contact with the aqueous environment, the gelatin completely dissolves due to the abundance of polar functional groups, such as amino, carboxyl, and hydroxyl groups. The presence of hydrogen bonds within these groups plays a key role in stabilizing the primary three-stranded helical structure of gelatin and its behavior under different conditions [30]. Therefore, in the present study, GA was used as a crosslinking agent to improve the mechanical properties of gelatin nanofibers. The underlying mechanism involves reducing the degree of gelatin hydrolysis through the interaction of GA with the hydrophilic regions of the protein. The basic principle of the crosslinking mechanism is based on the reaction of the carboxyl groups of GA and the numerous amino side groups of gelatin, forming the covalent amide bonds [31] (Scheme 1). Through the nucleophilic attack of the ϵ-amino groups of gelatin, further formation of strong covalent bonds leads to the renaturation of triple helical structures with a decrease in protein solubility (Figure 2).

On the other hand, crosslinking can cause negative consequences in terms of cytotoxicity and biocompatibility, especially at higher concentrations or in unreacted forms. Therefore, we optimized the crosslinking conditions by minimizing the GA concentration and using the vapor instead of direct exposure. The further removal of residual crosslinking agent content through vacuum conditions, as carried out in our study, is an additional step to ensure their safety for biomedical applications. Despite reports of cytotoxicity, the widespread success of numerous bioprosthetic implants proves the clinical acceptance of GA as a crosslinking agent [23]. Similarly, several studies have reported the successful crosslinking of gelatin nanofibrous scaffolds with GA as a crosslinking agent in biomedical applications [8,32,33].

Besides the presence of the mentioned crosslinking agent in nanofibers, the content of acetic acid, which is the main solvent, may affect several characteristics, including toxicity, degradation behavior, and biocompatibility. The goal of this study was to find and use the least toxic solvent feasible for the tissue-engineering domain. According to previous studies, acetic acid is a relatively good solvent for gelatin dissolving [34]. In particular, it was shown that elevated concentrations of acetic acid reduce surface tension, thereby improving the electrospinability, while at the same mitigating gelation phenomena through increased viscosity [35]. Despite the higher concentration, it is important to note that much of the acetic acid content evaporates during the electrospinning process as the nanofibers begin to solidify [36,37]. According to previously published studies, the pH values of gelatin scaffolds fabricated from acetic acid did not expose acidic properties in the physiological environment, which is essential for further cytotoxicity examination [38,39]. This conclusion follows the results presented in our study. As shown in Figure S1 (Supplementary Materials), the assessment of the pH values of crosslinked gelatin nanofibers (after 24 and 72 h) revealed pH changes that favor cell growth and proliferation under a physiological environment. There were no significant differences between antibiotic-free and antibiotic-incorporated gelatin scaffolds during the investigated time (24 and 72 h). Firstly, it could be explained by the composition of Dulbecco’s Eagle medium (DMEM), which contains sodium bicarbonate (NaHCO_3_), which represents a buffer agent that supports the maintenance of a stable pH system in controlled environment conditions such as we used in our study (5% CO_2_, 37 °C). Carbon dioxide (CO_2_) dissolves in the medium, forming carbonic acid (H_2_CO_3_), which dissociates into bicarbonate (HCO_3_^−^) and hydrogen ions (H^+^). The HCO_3_^−^ ions produced by the dissociation of H_2_CO_3_, as well as those produced by NaHCO_3_, contribute to the stability of the pH by buffering excess H^+^ ions.

On the other hand, the small concentrations of antibiotics in the scaffolds (0.1%) do not result in pH changes, which may additionally explain the environment’s constant pH value. Regarding the insignificant change in pH after 72 h (pH = 7.7 to 7.5), the hydrolysis of gelatin can explain these slight changes, as peptide bond hydrolysis and the release of the resulting oligopeptides with their free carboxylic groups can have an impact on the decreasing of the pH value.

After this step, the following assessment of the in vitro cytotoxicity was performed and proved the non-toxicity of the fabricated scaffolds (see Figure 8). Moreover, the proliferation of healthy MRC-5 cells increased in a time-dependent manner, showing an increased percentage of viable cells (under 80% after 72 h for all three types of scaffolds). Similarly, the results from literature data reported non-toxic effects of gelatin nanofibers constituted from acetic acid on healthy fibroblast L-929 cells [40]. It is important to note that the non-toxicity of acetic acid and GA in the cytotoxicity test was in accordance with the reported data [41,42], confirming the evaporation of acetic acid during the electrospinning process and the lack of inhibitory effects of residual GA in the nanofibers on cell activity.

Regarding the morphological characteristics of the nanofibers, the addition of antibiotics such as ciprofloxacin and gentamicin, dissolved in distilled water before being added to the gelatin solution, leads to a decrease in the electrical conductivity of the gelatin solution. This addition of dissolved antibiotics affects the concentration of acetic acid in the gelatin solution, thereby affecting its conductivity and viscosity. Consequently, these changes can affect the critical parameters of the electrospinning process. As a result, higher voltages and increased solution flow rates are often necessary, ultimately leading to the formation of larger nanofiber diameters [43]. As presented in Figure 3, the nanofibers containing antibiotics showed larger average diameters compared to the antibiotic-free scaffolds (with an average diameter (AD) of 425 ± 184 nm for antibiotic-free gelatin and 747 ± 86 nm and 571 ± 130 nm for nanofibers with ciprofloxacin and gentamicin, respectively). There was no significant difference observed in AD between gelatin/0.1% ciprofloxacin and gelatin/0.1% gentamicin nanofibers (747 ± 86 nm and 571 ± 130 nm). Our results are in line with previously published data, where the addition of ciprofloxacin into gelatin/PCL nanofibers increased the diameters of the nanofibers from initially 234 ± 98 nm to 518 ± 168 nm [44]. In another study, the incorporation of gentamicin into gelatin/PVA scaffolds also led to the average diameters increasing [45]. Also, it has been noted that crosslinking via GA influences the morphology of nanofibers, leading to larger diameters, which aligns with our outcomes [46,47]. Larger diameters, as demonstrated in early research, offer broad clinical application prospects in promoting tissue regeneration, which addresses the issue of insufficient blood supply during the early stages of the neoangiogenic process [48].

A thorough understanding of the molecular interactions, chemical composition, and spatial distribution of incorporated antibiotics within the gelatin scaffolds can be obtained by combining FTIR analysis and EDS mapping. Figure 4 shows the FTIR spectrum of the powder forms of antibiotics, crosslinked gelatin, and crosslinked gelatin nanofibers with loaded ciprofloxacin and gentamicin agents. Peaks that are characteristic of the gelatin structure were successfully obtained by FTIR spectrum: 1647 cm^−1^ for amide-I, 1537 cm^−1^ for amide-II, and 1243 cm^−1^ related to amide-III. Also, the groups’ shifting with the addition of GA suggests the existence of an intermolecular bond between gelatin and GA leading to the formation of aldimine linkage (C=N) (1450 cm^−1^). Nevertheless, no characteristic peaks of gentamicin and ciprofloxacin were observed on the obtained spectrum for gelatin/0.1% ciprofloxacin and gelatin/0.1% gentamicin nanofibers, possibly because of low amounts of antibiotics or because the characteristic peaks of antibiotics were covered by peaks of gelatin [49,50,51].

To prove that the ciprofloxacin and gentamicin were successfully loaded into the scaffolds, an EDS technique was used. The elemental distribution confirmed the presence of both antibiotic agents in gelatin nanofibers (Figure 5). This technique makes it possible to distinguish the structures of gelatin and gelatin incorporated with antibiotics, primarily by examining their different functional groups and molecular compositions. Gelatin, as a protein derivative, primarily consists of carbon (C), nitrogen (N), and oxygen (O) atoms. In contrast, antibiotics such as ciprofloxacin hydrochloride and gentamicin sulfate contain additional elements such as fluorine (F), chlorine (Cl), and sulfur (S), in addition to C, N, and O. The essential difference lies in the presence of these additional atoms in the antibiotic molecules. Our results indicate the presence of an elemental distribution which is consistent with the atomic compositions of ciprofloxacin hydrochloride and gentamicin sulfate. Therefore, it serves as compelling evidence for the successful incorporation of antibiotics into the gelatin scaffolds. Similarly, a previously published study reported the analogous tendency, where the presence of antibiotic agents was not detected by FTIR but determined using the EDS technique [22].

According to early research relatable to wound healing, the capacity to absorb and retain water is a key characteristic of wound-dressing materials [52]. The results of our study underline a high affinity for water due to the hydrophilic nature of gelatin molecules. As mentioned above in the results, we observed statistically significant (*p* < 0.05) results of water absorption for gelatin/0.1% ciprofloxacin (after 1 day = 657.89% and 7 days = 352.47%) compared with antibiotic-free gelatin (after 1 day = 566.66% and 7 days = 343.125%) and gelatin/0.1% gentamicin (after 1 day = 369.28% and 7 days = 132.45%) (Figure 6). Gelatin is a collagen-derived protein with a complex structure rich in amino acids including glycine, proline, and hydroxyproline. This structure enables gelatin to form a hydrogel with numerous polar groups that are capable of creating hydrogen bonds with water molecules, resulting in high water uptake. Gelatin is rich in hydrogen bond acceptor groups, primarily carbonyl groups (C=O). As water acts as a hydrogen bond donor, the interaction between the acceptor groups of gelatin and water molecules leads to enhanced water absorption.

The observation in our results for gelatin/0.1% gentamicin scaffolds can be explained by the chemical structure of gentamicin, which contains donor groups (-NH_2_ and -OH), while as noted, gelatin’s structure is abundant with carbonyl acceptor groups (C=O). In that manner, acceptor groups of gelatin will link with gentamicin due to the presence of donor groups, leaving fewer hydrogen-bonding sites for bonding with water [53]. Therefore, the possibilities for binding water are less, which is in accordance with the obtained results. Despite the reduced water absorption of gelatin/0.1% gentamicin scaffold (after 1 day = 369.28% and 7 days = 132.45%), it still exhibited a relatively high water retention capacity, allowing the absorption of wound exudate, making it a promising candidate for tissue-engineering applications. Furthermore, depending on the type of wound, the rapid absorption of water can sometimes hinder the healing process and increase the risk of additional infections and complications [54].

Regarding the gelatin/0.1% ciprofloxacin results, the highest rate of water absorption was related to the chemical structure of ciprofloxacin. It can be attributed to the abundance of acceptor groups in ciprofloxacin, including F, O, N, and COO^−^, facilitating fewer hydrogen bond interactions with gelatin, which is also rich in hydrogen acceptor groups. This in turn yields more hydrogen-bonding interactions with water and higher water uptake.

Similarly, we observed a statistically significant rate of degradation for gelatin/0.1% ciprofloxacin (21st day = 68.43%) compared with gelatin/0.1% gentamicin (21st day = 32.72%). Also, the rate of degradation for gelatin/0.1% ciprofloxacin was slightly faster than antibiotic-free gelatin (21st day = 59.27%). This is due to the relative water uptake, as gelatin/0.1% ciprofloxacin had the highest water uptake and faster degradation rate, followed by antibiotic-free gelatin, and lastly gelatin/0.1% gentamicin with its lowest water uptake had the lowest degradation rate (Figure 7).

Interestingly, the composition and structure of gelatin are responsible for its antibacterial properties. Protein residues from gelatin, including glycine, proline, and hydroxyproline, can damage bacterial cell walls and stop the growth of bacteria [55]. Moreover, the hydrophilic characteristics of gelatin can hinder bacterial adhesion at the wound site, helping to stop colonization and infection. The antibiotic-free gelatin scaffolds exhibited antimicrobial properties on both tested ATCC 25923 and ATCC 27853 bacteria (3.67 ± 0.47 and 2.00 ± 1.41, respectively).

There was no significant difference in antibacterial effects between gelatin/0.1% ciprofloxacin and gelatin/0.1% gentamicin aside from the fact gelatin/0.1% ciprofloxacin exposed the slightly better effect on ATCC 25923 bacteria (28.00 ± 3.74) (Table 1). Both antibiotic-loaded scaffolds exhibited better effects compared with the control (filter paper with antibiotics).

According to a previously published study, a nanofibrous membrane based on chitosan and polyvinyl alcohol gives a smaller diameter of inhibition than gelatin without antibiotics at *S. aureus* CMCC 26003. Also, nanofibrous membranes based on chitosan, polyvinyl alcohol, and ciprofloxacin hydrochloride, with and without graphene oxide, have a smaller inhibition diameter than gelatin without antibiotics. On the same bacteria, nanofibrous membranes based on chitosan, polyvinyl alcohol, and ciprofloxacin, with and without graphene oxide, also have a significantly smaller inhibition diameter (16.20 ± 1.21 mm and 15.30 ± 1.08 mm) than our gelatin with 0.1% ciprofloxacin [5]. Selected nanofibrous materials made of alginate, polyvinyl alcohol, and gelatin with the addition of ciprofloxacin show prominent activity against *S. aureus* ATCC 25923 [56]. The inhibition diameter of these two materials ranges from 10.52 mm and 20.97 mm, which is significantly less effective than gelatin with 0.1% ciprofloxacin (28.00 ± 3.74 mm).

The inherent properties of gelatin scaffolds, such as the capacity to facilitate cell adhesion and migration, release of angiogenic factors, control over the inflammatory response, and improved delivery of oxygen and nutrients, all contribute to the promotion of angiogenesis. The in vivo CAM assay represented the neoangiogenic potential of fabricated gelatin scaffolds. Increased vascularization of the surrounding tissue has been observed following the implantation of gelatin scaffolds in animal models, such as the chick chorioallantoic membrane. The ability of gelatin scaffolds to encourage the growth of new blood vessels in vivo is confirmed by this angiogenic response (Figure 9). Scaffolds containing antibiotic agents, such as gelatin/0.1% ciprofloxacin and gelatin/0.1% gentamicin, significantly stimulated the process of formation of new blood vessels (211.22%, and 274.58%, respectively) compared with the control (without treatment). However, the gelatin/0.1% gentamicin scaffolds showed a more pronounced angiogenic response (274.58%). Gelatin’s ability to control the release of cytokines that are both pro- and anti-inflammatory might promote angiogenesis by modifying the microenvironment. The gelatin scaffolds facilitate the migration of endothelial progenitor cells and other angiogenic cells to the wound site, thereby initiating the process of neovascularization by promoting a balanced inflammatory response [57].

Following the wound-healing treatment on rats, the application of gelatin/0.1% ciprofloxacin notably accelerated the regeneration compared to untreated wounds and wounds treated with gelatin/0.1% gentamicin scaffolds. Histological analysis revealed that gelatin/0.1% ciprofloxacin-treated wounds had prominent wound closure, less inflammation, and more collagen deposition than control wounds (Figure 10). Furthermore, ciprofloxacin’s antibacterial properties helped to prevent infections, making the environment more conducive to wound healing. Accordingly, the other study’s results observed that scaffolds with incorporated antibiotics could accelerate the wound-healing process [58]. Thus, the Chit/PEO/SiO_2_/ciprofloxacin scaffolds were noted as antibiotic-loaded nanofibers that noticeably accelerated the wound-healing process in rats compared with antibiotic-free Chit/PEO/SiO_2_/scaffold [59]. Another study discovered that incorporating ciprofloxacin into polyurethane-based hybrid scaffolds resulted in a proliferation of vascular endothelial cells around the wound site. This phenomenon facilitated the regeneration of blood capillaries, which consequently accelerated the healing process [60].

The results obtained in this study, suggest that the biomimetic ECM structure of gelatin nanofibers offers an environment suitable for activating material structure–intracellular signaling pathways. Furthermore, the addition of antibiotics has a dual function, improving both the structural integrity of the gelatin scaffolds and significantly contributing to the overall wound-healing process. Based on our knowledge, this is the first report assessing the potential of the GA-crosslinked electrospun gelatin nanofibrous mats with incorporated antibiotics used for neoangiogenesis.

## 4. Experimental

### 4.1. Materials and Methods

Gelatin (gel strength ~300 g Bloom, bovine skin, type A) and GA solution (Mw = 100.12 g/mol, 50% in H_2_O) were purchased from Sigma Aldrich (St. Louis, MO, USA). Dimethyl sulfoxide (DMSO, 99.5%), glacial acetic acid (AcA, 99.8%), and ethanol (96%) were from Fisher Chemical (Waltham, MA, USA). DMEM (high glucose, 4500 mg/L of glucose), 1% antibiotic agent penicillin/streptomycin, 10% fetal bovine serum (FBS), and 0.25% trypsin-EDTA were provided by Sigma Aldrich, while MTT (5 mg/mL, 98%) (3-[4,5-dimethylthiazol-2-yl]-2,5-diphenyltetrazolium bromide) was from Acros Organics (Geel, Belgium). PBS was obtained from Invitrogen Corporation, (Camarillo, CA, USA). The antibiotic agents ciprofloxacin hydrochloride and gentamicin sulfate were from Fujian Fukang Pharmaceutical, Fuzhou, China. The MRC-5 cell line was obtained from the American Tissue Culture Collection (Manassas, VA, USA). In this study, all chemicals were applied without further purification.

### 4.2. Fabrication of Gelatin Nanofibers

Five different combinations of gelatin solutions were investigated. The polymer-blend solutions were presented as gelatin in the AcA and DMSO co-solvent system with various ranges of concentration and volume ratios (Supplementary Materials). For further investigations, the most suitable solution for successful nanofiber production was determined to be 23% gelatin in a mixture of 70% AcA and 9% DMSO (93:7) (Table 2). To incorporate ciprofloxacin and gentamicin into the nanofibers, antibiotic powders were added as 0.1% content in *w*/*w* gelatin solution prior to the electrospinning.

The mixed solution was stirred using a magnetic stirrer (IKA Digital Color Squid, Fisher Scientific Waltham, MA, USA) at 40 °C for 1 h, and after that transferred to a 5 mL syringe with an 18-gauge needle. Subsequently, the syringe was then connected to a pump and the tip of the syringe was linked to the positive yield of a high-voltage source supplier (Acopian, Easton, PA, USA). Electrospinning was performed under controlled conditions set at 15 kV voltage and a flow rate of 0.15 mL/h. The distance between the tip of the needle and the collector was 15 cm. The nanofibers were collected on a collector plate covered with aluminum foil. The white aligned thin layer (the thickness is approximately 2 mm on the aluminum collector) of gelatin nanofibers was cut into 2 × 2 cm squares, placed in a dark place, and kept for further investigation.

### 4.3. Crosslinking of Nanofibers

Regarding gelatin structure, the electrospun scaffolds can immediately dissolve in an aqueous and physiological environment, losing the mechanical properties and stability of the fiber structure. Therefore, further improvement of wet resistance is urgently required before practical application. A common strategy involves the use of crosslinks to stabilize the scaffold structures. In this study, chemical crosslinking was carried out by exposing the scaffolds to saturated GA (25%) vapor for 6 h. After that, the samples were stored under vacuum conditions at room temperature for 120 h to remove the residual solvent content.

#### 4.3.1. pH Evaluation

The pH values of gelatin scaffolds, especially those produced using acetic acid as a solvent, can have a noticeable effect on cells due to their acidic nature. Acetic acid, as a weak acid, can affect the pH of the scaffold material and consequently affect cell behavior and viability. Thus, the pH of the medium (DMEM) containing the gelatin scaffolds was carefully evaluated. The color changes in the medium after exposure to electrospun gelatin fibers fabricated from acid content were investigated after 24 and 72 h in physiological conditions at 37 °C 5% CO_2_. In addition, the effect of the incorporation of antibiotics such as ciprofloxacin and gentamicin into gelatin fibers on the pH medium was also examined. The evaluation was carried out using a digital pH meter (AL10PH HAND-HELD-METER, Roth, Germany) with average values obtained from at least three measurements for each solution.

#### 4.3.2. Characterization and Morphology of Nanofibrous Mats

The initial morphologies and diameters of obtained nanofibers were observed with both an inverted microscope (Delta Optical Genetic PRO microscope, Warsaw, Poland) and a scanning electron microscope (SEM) (MIRA3 TESCAN, Brno, Czech Republic). Fiber diameters were determined by analyzing five SEM images using the Image J Software Package (version 1.48). At least 100 measures per micrograph were taken to estimate average fiber diameters. The results were recorded in MS Excel 2016 (Version 2108) and expressed as the mean of value ± standard error (Table 2). In addition, the diameter distribution for all five combinations is presented in the Supplementary Materials.

#### 4.3.3. SEM and EDS Analysis

Before the observations, the samples were cut into small squares, sputter-coated with gold, and analyzed with a scanning electron microscope. The SEM investigations were conducted at various magnifications, with the acceleration voltage at 15 kV for 3 min. SEM equipped with an EDS detector was used to determine the elemental distribution of ciprofloxacin and gentamicin in the gelatin nanofibers.

#### 4.3.4. FTIR Spectroscopy

The chemical structure and conformation of the gelatin scaffolds were analyzed by transmission infrared spectroscopy (portable FTIR/FT-NIR spectrometer Interspec 301-X, Toravere Estonia) in the spectral range between 600 and 4000 cm^−1^, with 20 scans per specimen, in the resolution of 2 cm^−1^. The powdered samples were prepared by mixing with KBr in a 1:100 ratio, while electrospun fibers were examined in electrospun form.

#### 4.3.5. Cytotoxicity Assay

The designed nanofibrous antibiotic-free gelatin, gelatin/0.1% ciprofloxacin, and gelatin/0.1% gentamicin scaffolds were assessed for in vitro cytotoxicity by the viability of MRC-5 healthy fibroblast cells from pleura by MTT assay (Laboratory for Bioengineering, Kragujevac, Serbia). Briefly, the scaffolds were sterilized under UV light for 30 min and accurately placed onto each bottom of a 96-well plate. The cells were cultured in DMEM at 37 °C under a 5% CO_2_ and 95% humidity atmosphere for 24 h. Before the experiment, the scaffolds were accurately cut into small pieces and placed onto each bottom. After that, the 100 μL of cell suspension was added to the surface of nanofibrous mats and the plate was incubated for 24 h for cell attachment. Fresh culture medium was used as a blank. Cell viability was monitored at 24 and 72 h. The optical density of the purple formazan solution, indicating cell viability, was quantified using a Multiskan SkyHigh Microplate Spectrophotometer (ThermoFisher Scientific, Inc., Waltham, MA, USA) at a wavelength of 492 nm. Each sample was analyzed in triplicate, and the data expressed as mean ± standard error.

#### 4.3.6. Test of Absorption and Degradation

The water absorption and degradation of scaffolds are the essential properties for a successful skin-regeneration process. For both tests, the samples were cut (2.5 × 2.5 cm), weighed, and placed into a 6-well plate in 4 mL PBS (pH = 7.4) and incubated under physiological conditions (37 °C, 5% CO_2_). For 7 days, every 24 h, the scaffolds were taken out, wiped with filter paper to remove excess water, dried in a vacuum at room temperature, and weighed. Water uptake was calculated using the following formula:

(1) $$Water\;uptake\left(\%\right)=\frac{W_{w}-W_{d}}{W_{d}}\times 100\%$$

where *W_d_* is the weight of the dry sample, and *W_w_* is the wet weight after a different time interval (every day, during the 7 days) in PBS.

Degradation mass (%) was calculated using a specific equation:

(2) $$Degradation\;mass(\%)=\frac{W_{t}}{W_{0}}\times 100\%$$

where *W*_0_ is the weight in dry conditions, after vacuum drying, and *W_t_* is the weight of the degraded sample. All experiments were performed in duplicate.

#### 4.3.7. Antibacterial Activity

The antibacterial activity of antibiotic-free gelatin, gelatin/0.1% ciprofloxacin, and gelatin/0.1% gentamicin scaffolds was tested against two standard strains of bacteria. Filter papers with gentamicin and ciprofloxacin (1 mg/mL) were used as positive controls. The experiment involved one Gram-positive bacterium (*Staphylococcus aureus* ATCC 25923) and one Gram-negative bacterium (*Pseudomonas aeruginosa* ATCC 27853).

##### Preparation of Bacterial Suspensions and Tested Materials

Bacterial cultures were first cultivated on nutrient agar and then incubated for 18–20 h at 37 °C to ensure optimal growth conditions. Bacterial suspensions were then prepared by the direct colony method, with suspension turbidity adjusted to McFarland 0.5 corresponding to 10^8^ CFU/mL using a densitometer (DEN-1, BioSan, Riga, Latvia). These suspensions were prepared immediately before the experiment, as they should be used approximately within 15 min of preparation [61].

Gelatin scaffolds were cut into cylinders with a diameter of 8 mm, while filter paper with a diameter of 5 mm was used. In particular, the scaffold material was not taken into account when measuring the inhibition diameter.

##### Disk-Diffusion Susceptibility Test

The susceptibility of bacteria to the tested films and standard antibiotics was tested by the in vitro disk diffusion method. The disk diffusion test was performed in a Petri dish on Mueller–Hinton agar (25 mL of medium per plate). Gelatin scaffolds both with and without antibiotics, along with filter paper discs containing antibiotics, were placed on the surface of the medium (3 identical scaffolds/papers on 1 plate), on which a pure bacterial suspension with 1–2 × 10^8^ CFU/mL was cultivated. After incubation (16–24 h), the inhibition zone diameter (the surface of the bacterial growth inhibition zone) was measured. The obtained values were compared with the EUCAST standard. All zones of inhibition were calculated in triplicate.

#### 4.3.8. Chick Embryo Chorioallantoic Membrane (CAM) Assay

**Figure 11:**
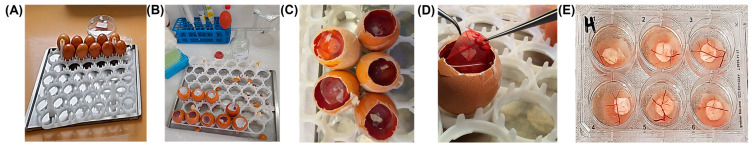
The CAM assessment with the following steps: (**A**) collection, preparation, and disinfection of eggs using 10% iodine solution, (**B**) a precise cut made on the eggshell, (**C**) implantation of scaffolds in the chicken embryo membrane, (**D**) excision of the implanted area after the stated incubation time, and (**E**) preparation of a 6-well plate with embedded samples for microscopic analysis.

In our research, we used the CAM assay to investigate how effectively gelatin scaffolds promote the growth of new blood vessels. First, we collected 40 fertilized chicken eggs and subjected them to a five-week incubation period at 38 °C and 60% humidity. The 10-day-old embryos were then randomly assigned to one of four experimental groups, each containing ten eggs: control group, antibiotic-free gelatin, gelatin/0.1% ciprofloxacin, and gelatin/0.1% gentamicin scaffolds. Figure 11 represents the steps of the procedure.

During the incubation period, the development and morphology of the eggs were carefully monitored using an egg candle. Before starting the experiment, we sterilized the egg surfaces with a 10% iodine solution in a laminar airflow environment.

After sterilization, a precise incision was made in the eggshell using aseptic techniques to expose the inner membrane underneath. Sterilized scaffolds were then placed on top of the inner membrane. In addition, we included six sealed eggs in the control group to monitor for possible contamination. The exposed areas of the eggs were covered with parafilm, and the eggs were placed in an incubator at 37 °C for five days until day 15 of fertilization. During the experimental period, we performed daily checks to detect signs of contamination and monitor embryo growth. At the end of the incubation period, we removed the parafilm and cleaned the membranes with 1 mL of 4% paraformaldehyde (PFA). The membrane encapsulated in the scaffold was carefully excised and transferred to a 6-well plate containing PBS. All embryos used for the treatment were humanely sacrificed.

Ex-ovo imaging was performed directly from the 6-well plate using a light microscope (Delta Optical Genetic PRO, Poland). We quantified neoangiogenesis by counting vessels associated with the gelatin scaffolds using ImageJ software. The obtained data are expressed as percentage values to facilitate comparative analysis.

#### 4.3.9. In Vivo Wound Healing

In this study, the male Wistar albino rats (210–270 g) used were six to eight weeks old. The animals were obtained from the Military Medical Academy, Belgrade, Serbia, and were kept in transparent cages with ad libitum access to water and food at the temperature of 22 ± 2 °C, with a 12/12 h light/dark cycle. All procedures were completed according to EU Directive 86/609/EEC on the welfare of laboratory animals and the principles of Good Laboratory Practice (GLP). The animals were randomly divided into five groups (3 animals per group).

1. Negative control—healthy skin from the dorsal middle back of rats;
2. Positive control group—rats were subjected to burn wounds without any further treatment, serving as the control for the experiment;
3. Antibiotic-free gelatin—burn wounds on rats were treated with antibiotic-free gelatin;
4. Gelatin/0.1% ciprofloxacin scaffolds—burn wounds on rats were treated with scaffolds containing gelatin/0.1% ciprofloxacin;
5. Gelatin/0.1% gentamicin scaffolds—burn wounds on rats were treated with scaffolds containing gelatin/0.1% gentamicin.

##### Experimental Model of Burn Wounds

To induce burn wounds, animals were anesthetized with a mixture of ketamine (5 mg/kg) and xylazine (10 mg/kg) intraperitoneally. Primarily, the procedure involved shaving the rats’ backs and preparing the animals for the procedure. Creating full-thickness burn wounds (2 × 2 cm) was performed by applying a hot aluminum plate to the skin on the dorsal middle part of the back for 10 s as previously described in [62]. The burn wounds were then covered with the previously prepared scaffolds (2 × 2 cm), and the healing development was observed for seven days, with daily scaffold replacements.

##### Histopathological Analysis

In order to evaluate the skin regeneration of rats as a response to the experimental treatments, histopathological analysis was carried out. On the 7th day after the induction of burn wounds, all the animals were sacrificed through cervical dislocation in short-term ketamine/xylazine anesthesia. The wounds of all animals were dissected with a margin of healthy skin around the boundaries of the wounds and fixed in a 10% buffered-formalin fixative solution overnight. Subsequently, all the tissue samples were undertaken in the process of dehydration through successive alcohol concentration increases, and in the end, the tissue samples were embedded in paraffin. The paraffin blocks were cut into 5 µm sections, prepared, and stained with hematoxylin and eosin (H&E). Following that, the stained slides were observed and photographed under a light microscope (Olympus BX, Tokyo, Japan) with a digital camera to evaluate the level of tissue damage and regeneration.

#### 4.3.10. Ethics Approval

The pretreatment and experimental procedures were performed following European Directive 2010/63/EU on the welfare of laboratory animals, following approval by the Ethical Committee of the Faculty of Medical Sciences, University of Kragujevac, Serbia (Study Nr. 01-6121 from 27.07.2020).

#### 4.3.11. Statistical Analysis

All data are presented as mean value ± standard error (SE). A one-way ANOVA test was used to determine statistical significance. The magnitude of correlation between variables was calculated using the SPSS statistical software package (Chicago, IL, USA) (SPSS for Windows, ver.17, 2008).

## 5. Conclusions

This study reports on the development of a simple and effective process for creating artificial biomaterials. Gelatin nanofibers crosslinked with GA vapor were successfully fabricated and investigated by in vitro and in vivo methods. The fundamental aim of this study was to synthesize a natural biomaterial that is not only biocompatible and non-toxic but also applicable for use in wound-healing treatments. The findings highlight the importance of gelatin’s structural similarity to the extracellular matrix environment. Cytotoxicity test results revealed that nanofibrous gelatin scaffolds promoted the proliferation of healthy cells in a time-dependent manner, especially when treated with antibiotic-loaded gelatin scaffolds. Moreover, the antibacterial properties of the antibiotic-free gelatin scaffolds underline the innate ability of gelatin to fight bacteria without the addition of antibiotics. The SEM, FTIR, and EDS analysis proved the aligned morphology, proper chemical structure, and successful antibiotic incorporation.

The rate of water absorption indicated that gelatin scaffolds can absorb large amounts of exudate, while degradation under physiological conditions demonstrated the biodegradability and preferable properties of the native gelatin structure, which is important for successful wound healing. Finally, the in vivo experiments performed on a rat model indicated an accelerated epithelialization of the skin, especially with the addition of ciprofloxacin, highlighting the potential of gelatin scaffolds with their pro-angiogenic activity, which was additionally proved by a CAM assay.

In summary, our study revealed that antibiotic-incorporated gelatin scaffolds promote wound healing by providing structural support, preventing infection, and stimulating the neoangiogenesis process. This dual functionality favors prominent wound closure and tissue regeneration.

## Figures and Tables

**Table 1:** Zone diameter interpretation results of tested materials with disk diffusion method for tested bacteria.

Tested Bacteria/ Tested Material	*Staphylococcus aureus* ATCC 25923	*Pseudomonas aeruginosa* ATCC 27853
Antibiotic-free gelatin scaffold	3.67 ± 0.47 *	2.00 ± 1.41
Gelatin/0.1% gentamicin scaffold	21.67 ± 1.25	22.33 ± 1.70
Filter paper/gentamicin 1 mg/mL	20.00 ± 2.16	14.33 ± 3.40
Gelatin/0.1% ciprofloxacin scaffold	28.00 ± 3.74	18.33 ± 4.11
Filter paper/ciprofloxacin 1 mg/mL	27.33 ± 1.25	25.33 ± 0.47

* The inhibition zone diameter is given in mm without the surface of the tested material.

**Table 2:** Parameters of electrospinning process and diameter properties of non-crosslinked nanofibers.

Solution Parameters		Electrospinning Conditions					
Gelatin Concentration	AcA/DMSO (*v*/*v*%)	Voltage (kV)	Flow Rate (mL/h)	Nozzle Size (gauge)	Collector and Needle Distance (cm)	Temperature and Humidity Conditions	Average Diameter (nm)
23%	70% AcA: 9% DMSO (93:7)	15	0.15	18	15	28 °C, 45%	376 ± 122

## Data Availability

The authors declare that all data supporting the findings are available within the paper or are available from the authors upon request.

## Supplementary Materials

The following supporting information can be downloaded at: https://www.mdpi.com/article/10.3390/ph17070851/s1, Figure S1: pH evaluation of scaffolds after 24 and 72 h in the medium. A1—Antibiotic-free gelatin, A2—gelatin/0.1% ciprofloxacin, and A3—gelatin/0.1% gentamicin scaffolds. B1, B2, B3—Control medium; Figure S2: Fiber Diameter Distribution Analysis of Gelatin Electrospun nanofibers for Series 1: 16-24% Gelatin in 99.8% AcA: 9% DMSO (93:7); Figure S3: Fiber Diameter Distribution Analysis of Gelatin Electrospun nanofibers for Series 2: 18–30% Gelatin in 70% AcA: 9% DMSO (93:7); Figure S4: Fiber Diameter Distribution Analysis of Gelatin Electrospun nanofibers for Series 3: 18-30% Gelatin in 70% AcA: 9% DMSO (97:3); Figure S5: Fiber Diameter Distribution Analysis of Gelatin Electrospun nanofibers for Series 3: 19-31% Gelatin in 70% AcA: 9% DMSO (91:9); Figure S6: Fiber Diameter Distribution Analysis of Gelatin Electrospun nanofibers for Series 5: 19-31% Gelatin in 70% AcA: 9% DMSO (95:5); Figure S7: Non-crosslinked gelatin fibers (a) and GA-crosslinked gelatin fibers (b); Figure S8—An example of the action of gelatin scaffold: (a) *Staphylococcus aureus* ATCC 25923—gelatin/0.1% gentamicin scaffold (b) *S. aureus* ATCC 25923—gelatin/0.1% ciprofloxacin scaffold (c) *Pseudomonas aeruginosa* ATCC 27853—gelatin/0.1% gentamicin scaffold and (d) *P. aeruginosa* ATCC 27853—gelatin/0.1% ciprofloxacin scaffold; Figure S9: An example of the action of gelatin scaffold: (a) *Staphylococcus aureus* ATCC 25923—filter paper/gentamicin 1 mg/mL (b) *S. aureus* ATCC 25923—filter paper/ciprofloxacin 1 mg/mL (c) *Pseudomonas aeruginosa* ATCC 27853—filter paper/gentamicin 1 mg/mL and (d) *P. aeruginosa* ATCC 27853—filter paper/ciprofloxacin 1 mg/mL.

## References

[1] Li T., Sun M., Wu S. (2022). State-of-the-Art Review of Electrospun Gelatin-Based Nanofiber Dressings for Wound Healing Applications. Nanomaterials.

[2] Augustine R., Kalarikkal N., Thomas S. (2014). Advancement of Wound Care from Grafts to Bioengineered Smart Skin Substitutes. Prog. Biomater..

[3] Chinnappan B.A., Krishnaswamy M., Xu H., Hoque M.E. (2022). Electrospinning of Biomedical Nanofibers/Nanomembranes: Effects of Process Parameters. Polymers.

[4] Liu S., Yu J.-M., Gan Y.-C., Qiu X.-Z., Gao Z.-C., Wang H., Chen S.-X., Xiong Y., Liu G.-H., Lin S.-E. (2023). Biomimetic Natural Biomaterials for Tissue Engineering and Regenerative Medicine: New Biosynthesis Methods, Recent Advances, and Emerging Applications. Mil. Med. Res..

[5] Cui C., Sun S., Wu S., Chen S., Ma J., Zhou F. (2021). Electrospun Chitosan Nanofibers for Wound Healing Application. Eng. Regen..

[6] Vasita R., Katti D.S. (2006). Nanofibers and Their Applications in Tissue Engineering. Int. J. Nanomed..

[7] Chong E., Phan T., Lim I., Zhang Y., Bay B., Ramakrishna S., Lim C. (2007). Evaluation of Electrospun PCL/Gelatin Nanofibrous Scaffold for Wound Healing and Layered Dermal Reconstitution. Acta Biomater..

[8] Sabzi M., Pirmoradian A., Behshad Y., Sharifi F., Alipour S., Jiang L. (2023). Development of Antibiotic Releasing Electrospun Nanofibrous Mats Based on Gelatin. Mater Chem Horiz..

[9] Mozaffari A., Parvinzadeh M. (2022). Effect of Voltage and Distance in Electrospinning of Gelatin. Iran. J. Text. Nano-Bio Modif..

[10] Afewerki S., Sheikhi A., Kannan S., Ahadian S., Khademhosseini A. (2019). Gelatin-polysaccharide Composite Scaffolds for 3D Cell Culture and Tissue Engineering: Towards Natural Therapeutics. Bioeng. Transla Med..

[11] Sharma P.C., Jain A., Jain S., Pahwa R., Yar M.S. (2010). Ciprofloxacin: Review on Developments in Synthetic, Analytical, and Medicinal Aspects. J. Enzym. Inhib. Med. Chem..

[12] Xia L., Lu L., Liang Y., Cheng B. (2019). Fabrication of Centrifugally Spun Prepared Poly(Lactic Acid)/Gelatin/Ciprofloxacin Nanofibers for Antimicrobial Wound Dressing. RSC Adv..

[13] Thy M., Timsit J.-F., De Montmollin E. (2023). Aminoglycosides for the Treatment of Severe Infection Due to Resistant Gram-Negative Pathogens. Antibiotics.

[14] López-Calderón H.D., Avilés-Arnaut H., Galán-Wong L.J., Almaguer-Cantú V., Laguna-Camacho J.R., Calderón-Ramón C., Escalante-Martínez J.E., Arévalo-Niño K. (2020). Electrospun Polyvinylpyrrolidone-Gelatin and Cellulose Acetate Bi-Layer Scaffold Loaded with Gentamicin as Possible Wound Dressing. Polymers.

[15] Ehrmann A. (2021). Non-Toxic Crosslinking of Electrospun Gelatin Nanofibers for Tissue Engineering and Biomedicine—A Review. Polymers.

[16] Lu W., Ma M., Xu H., Zhang B., Cao X., Guo Y. (2015). Gelatin Nanofibers Prepared by Spiral-Electrospinning and Cross-Linked by Vapor and Liquid-Phase Glutaraldehyde. Mater. Lett..

[17] Chen H., Jao W., Yang M. (2009). Characterization of Gelatin Nanofibers Electrospun Using Ethanol/Formic Acid/Water as a Solvent. Polym. Adv. Techs.

[18] Pal A., Kumar Bajpai A., Bajpai J. (2019). Study on Facile Designing, Swelling Properties and Structural Relationship of Gelatin Nanoparticles. J. Macromol. Sci. Part A.

[19] Lin J., Pan D., Sun Y., Ou C., Wang Y., Cao J. (2019). The Modification of Gelatin Films: Based on Various Cross-linking Mechanism of Glutaraldehyde at Acidic and Alkaline Conditions. Food Sci. Nutr..

[20] Dwivedi C., Pandey H., Pandey A., Ramteke P. (2015). Fabrication and Assessment of Gentamicin Loaded Electrospun Nanofibrous Scaffolds as a Quick Wound Healing Dressing Material. Curr. Nanosci..

[21] Silva D.M., Vyas H.K.N., Sanderson-Smith M.L., Sencadas V. (2018). Development and Optimization of Ciprofloxacin-Loaded Gelatin Microparticles by Single-Step Spray-Drying Technique. Powder Technol..

[22] Lin M., Liu Y., Gao J., Wang D., Xia D., Liang C., Li N., Xu R. (2022). Synergistic Effect of Co-Delivering Ciprofloxacin and Tetracycline Hydrochloride for Promoted Wound Healing by Utilizing Coaxial PCL/Gelatin Nanofiber Membrane. Int. J. Mol. Sci..

[23] Bigi A., Cojazzi G., Panzavolta S., Rubini K., Roveri N. (2001). Mechanical and Thermal Properties of Gelatin Films at Different Degrees of Glutaraldehyde Crosslinking. Biomaterials.

[24] EUCAST—European Committee on Antimicrobial Susceptibility Testing Breakpoint Tables for Interpretation of MICs and Zone Diameters. Version 14.0. https://www.eucast.org.

[25] Salimi F., Mohammadipanah F. (2021). Nanomaterials Versus The Microbial Compounds With Wound Healing Property. Front. Nanotechnol..

[26] Anjum S., Rahman F., Pandey P., Arya D.K., Alam M., Rajinikanth P.S., Ao Q. (2022). Electrospun Biomimetic Nanofibrous Scaffolds: A Promising Prospect for Bone Tissue Engineering and Regenerative Medicine. Int. J. Mol. Sci..

[27] Sell S.A., Wolfe P.S., Garg K., McCool J.M., Rodriguez I.A., Bowlin G.L. (2010). The Use of Natural Polymers in Tissue Engineering: A Focus on Electrospun Extracellular Matrix Analogues. Polymers.

[28] Lazaridou M., Bikiaris D.N., Lamprou D.A. (2022). 3D Bioprinted Chitosan-Based Hydrogel Scaffolds in Tissue Engineering and Localised Drug Delivery. Pharmaceutics.

[29] Lazaridou M., Moroni S., Klonos P., Kyritsis A., Bikiaris D.N., Lamprou D.A. (2024). 3D-Printed Hydrogels Based on Amphiphilic Chitosan Derivative Loaded with Levofloxacin for Wound Healing Applications. Int. J. Polym. Mater. Polym. Biomater..

[30] Silva R.S.G., Bandeira S.F., Pinto L.A.A. (2014). Characteristics and Chemical Composition of Skins Gelatin from Cobia (Rachycentron Canadum). LWT—Food Sci. Technol..

[31] Campiglio C.E., Contessi Negrini N., Farè S., Draghi L. (2019). Cross-Linking Strategies for Electrospun Gelatin Scaffolds. Materials.

[32] Chen W., Gao Z., He M., Dou Y., Yin G., Ding J. (2022). Vapor-Phase Glutaraldehyde Crosslinked Waste Protein-Based Nanofiber Nonwovens as an Environmentally Friendly Wound Dressing. React. Funct. Polym..

[33] Banafati Zadeh F., Zamanian A. (2022). Glutaraldehyde: Introducing Optimum Condition for Cross-Linking the Chitosan/Gelatin Scaffolds for Bone Tissue Engineering. Int. J. Eng..

[34] Aoki H., Miyoshi H., Yamagata Y. (2015). Electrospinning of Gelatin Nanofiber Scaffolds with Mild Neutral Cosolvents for Use in Tissue Engineering. Polym. J..

[35] Avossa J., Herwig G., Toncelli C., Itel F., Rossi R.M. (2022). Electrospinning Based on Benign Solvents: Current Definitions, Implications and Strategies. Green Chem..

[36] Chi H.Y., Chang N.Y., Li C., Chan V., Hsieh J.H., Tsai Y.-H., Lin T. (2022). Fabrication of Gelatin Nanofibers by Electrospinning—Mixture of Gelatin and Polyvinyl Alcohol. Polymers.

[37] Abdulkadhim M.K., Habeeb S.A. (2023). Electro Spun Uniform Nanofiber from Gelatin: Chitosan at Low Concentration. Mater. Today Proc..

[38] Erencia M., Cano F., Tornero J.A., Fernandes M.M., Tzanov T., Macanás J., Carrillo F. (2015). Electrospinning of Gelatin Fibers Using Solutions with Low Acetic Acid Concentration: Effect of Solvent Composition on Both Diameter of Electrospun Fibers and Cytotoxicity. J. Appl. Polym. Sci..

[39] Binulal N.S., Natarajan A., Menon D., Bhaskaran V.K., Mony U., Nair S.V. (2014). PCL–Gelatin Composite Nanofibers Electrospun Using Diluted Acetic Acid–Ethyl Acetate Solvent System for Stem Cell-Based Bone Tissue Engineering. J. Biomater. Sci. Polym. Ed..

[40] Jalaja K., James N.R. (2015). Electrospun Gelatin Nanofibers: A Facile Cross-Linking Approach Using Oxidized Sucrose. Int. J. Biol. Macromol..

[41] Campiglio C.E., Ponzini S., De Stefano P., Ortoleva G., Vignati L., Draghi L. (2020). Cross-Linking Optimization for Electrospun Gelatin: Challenge of Preserving Fiber Topography. Polymers.

[42] Zhang Y.Z., Venugopal J., Huang Z.-M., Lim C.T., Ramakrishna S. (2006). Crosslinking of the Electrospun Gelatin Nanofibers. Polymer.

[43] Deng L., Taxipalati M., Zhang A., Que F., Wei H., Feng F., Zhang H. (2018). Electrospun Chitosan/Poly(Ethylene Oxide)/Lauric Arginate Nanofibrous Film with Enhanced Antimicrobial Activity. J. Agric. Food Chem..

[44] Ajmal G., Bonde G.V., Mittal P., Khan G., Pandey V.K., Bakade B.V., Mishra B. (2019). Biomimetic PCL-Gelatin Based Nanofibers Loaded with Ciprofloxacin Hydrochloride and Quercetin: A Potential Antibacterial and Anti-Oxidant Dressing Material for Accelerated Healing of a Full Thickness Wound. Int. J. Pharm..

[45] Cesur S., Ilhan E., Tut T.A., Kaya E., Dalbayrak B., Bosgelmez-Tinaz G., Arısan E.D., Gunduz O., Kijeńska-Gawrońska E. (2023). Design of Cinnamaldehyde- and Gentamicin-Loaded Double-Layer Corneal Nanofiber Patches with Antibiofilm and Antimicrobial Effects. ACS Omega.

[46] Okutan N., Terzi P., Altay F. (2014). Affecting Parameters on Electrospinning Process and Characterization of Electrospun Gelatin Nanofibers. Food Hydrocoll..

[47] Baykara D., Pilavci E., Cesur S., Ilhan E., Ulag S., Sengor M., Kijeńska-Gawrońska E., Gunduz O. (2023). Controlled Release of Gentamicin from Electrospun Poly(Vinyl Alcohol)/Gelatin Nanofibers: The Effect of Crosslinking Time Using Glutaraldehyde Vapor. ChemistrySelect.

[48] Liu Y., Wang X., Hu F., Rausch-Fan X., Steinberg T., Lan Z., Zhang X. (2022). The Effect of Modifying the Nanostructure of Gelatin Fiber Scaffolds on Early Angiogenesis in Vitro and in Vivo. Biomed. Mater..

[49] Masoumi S., Amiri S., Bahrami S.H. (2018). PCL-Based Nanofibers Loaded with Ciprofloxacin/Cyclodextrin Containers. J. Text. Inst..

[50] Cui C., Sun S., Li X., Chen S., Wu S., Zhou F., Ma J. (2022). Optimizing the Chitosan-PCL Based Membranes with Random/Aligned Fiber Structure for Controlled Ciprofloxacin Delivery and Wound Healing. Int. J. Biol. Macromol..

[51] Wang Q., Dong Z., Du Y., Kennedy J.F. (2007). Controlled Release of Ciprofloxacin Hydrochloride from Chitosan/Polyethylene Glycol Blend Films. Carbohydr. Polym..

[52] Nuutila K., Eriksson E. (2021). Moist Wound Healing with Commonly Available Dressings. Adv. Wound Care.

[53] Zhan J., Morsi Y., Ei-Hamshary H., Al-Deyab S.S., Mo X. (2016). In Vitro Evaluation of Electrospun Gelatin–Glutaraldehyde Nanofibers. Front. Mater. Sci..

[54] Liang Y., Liang Y., Zhang H., Guo B. (2022). Antibacterial Biomaterials for Skin Wound Dressing. Asian J. Pharm. Sci..

[55] Madkhali O.A. (2023). Drug Delivery of Gelatin Nanoparticles as a Biodegradable Polymer for the Treatment of Infectious Diseases: Perspectives and Challenges. Polymers.

[56] Thairin T., Wutticharoenmongkol P. (2022). Ciprofloxacin-Loaded Alginate/Poly (Vinyl Alcohol)/Gelatin Electrospun Nanofiber Mats as Antibacterial Wound Dressings. J. Ind. Text..

[57] Dreesmann L., Ahlers M., Schlosshauer B. (2007). The Pro-Angiogenic Characteristics of a Cross-Linked Gelatin Matrix. Biomaterials.

[58] Negut I., Dorcioman G., Grumezescu V. (2020). Scaffolds for Wound Healing Applications. Polymers.

[59] Hashemikia S., Farhangpazhouh F., Parsa M., Hasan M., Hassanzadeh A., Hamidi M. (2021). Fabrication of Ciprofloxacin-Loaded Chitosan/Polyethylene Oxide/Silica Nanofibers for Wound Dressing Application: In Vitro and in Vivo Evaluations. Int. J. Pharm..

[60] Li H., Williams G.R., Wu J., Lv Y., Sun X., Wu H., Zhu L.-M. (2017). Thermosensitive Nanofibers Loaded with Ciprofloxacin as Antibacterial Wound Dressing Materials. Int. J. Pharm..

[61] Andrews J.M. (2005). BSAC Standardized Disc Susceptibility Testing Method (Version 4). J. Antimicrob. Chemother..

[62] Qu J., Zhao X., Liang Y., Zhang T., Ma P.X., Guo B. (2018). Antibacterial Adhesive Injectable Hydrogels with Rapid Self-Healing, Extensibility and Compressibility as Wound Dressing for Joints Skin Wound Healing. Biomater.

