# Supplementary material for: Electrospun Gelatin Scaffolds with Incorporated Antibiotics for Skin Wound Healing
Source: Pharmaceuticals (Basel). 2024 Jun 28;17(7):851. doi: 10.3390/ph17070851 (PMC11280474; doi:10.3390/ph17070851)
Supplement: Supplementary file 1 [file pharmaceuticals-17-00851-s001.zip › pharmaceuticals-3044691-supplementary.pdf]

Electrospun Gelatin Scaffolds with Incorporated Antibiotics for Skin Wound Healing

Katarina Virijević[1*], Marko Živanović[1], Jelena Pavić[1], Luka Dragačević[2], Biljana Ljujić[3], Marina Miletić Kovačević[4], Miloš Papić[5], Suzana Živanović[5], Strahinja Milenković[6], Ivana Radojević[7] and Nenad Filipović[6,8]

[1] Institute for Information Technologies, University of Kragujevac, Kragujevac 34000, Serbia

[2] Institute of Virology, Vaccines and Sera "Torlak", Belgrade 11000, Serbia

[3] Department of Genetics, Faculty of Medical Sciences, University of Kragujevac, Kragujevac 34000, Serbia

[4] Department of Histology and Embryology, Faculty of Medical Sciences, University of Kragujevac, Kragujevac 34000, Serbia

[5] Department of Dentistry, Faculty of Medical Sciences, University of Kragujevac, Kragujevac 34000, Serbia

[6] Faculty of Engineering, University of Kragujevac, Kragujevac 34000, Serbia

[7] Faculty of Natural Sciences, Department of Biology and Ecology, Kragujevac 34000, Serbia

[8] BioIRC - Bioengineering Research and Development Center, Kragujevac 34000, Serbia

* Corresponding author: Katarina Virijević

Email: katarina.virijevic@uni.kg.ac.rs

pH evaluation of gelatin scaffolds in a physiological environment (37°C, 5% CO_2)

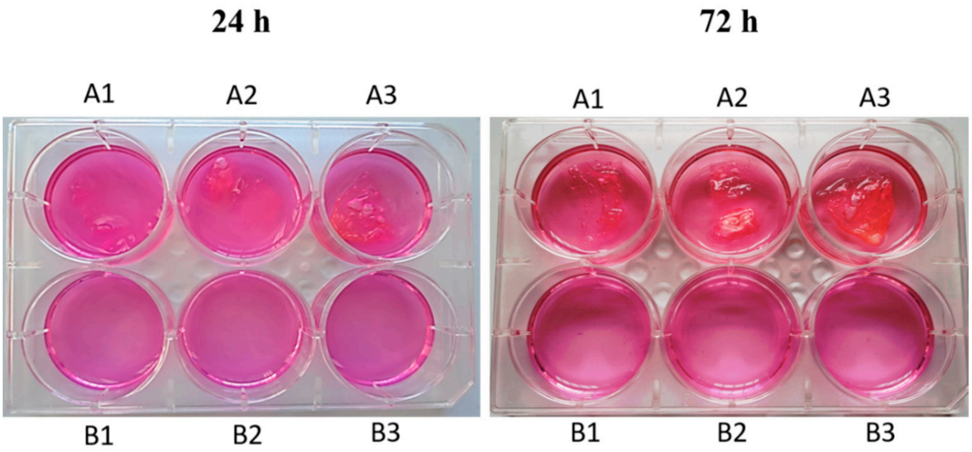

Figure S1. pH evaluation of scaffolds after 24 and 72 hours in the medium; A1– Antibiotic-free gelatin, A2 – gelatin/0.1% ciprofloxacin, and A3 – gelatin/0.1% gentamicin scaffolds; B1, B2, B3 – Control medium.

Diameter distribution of nanofibers for each polymer combination

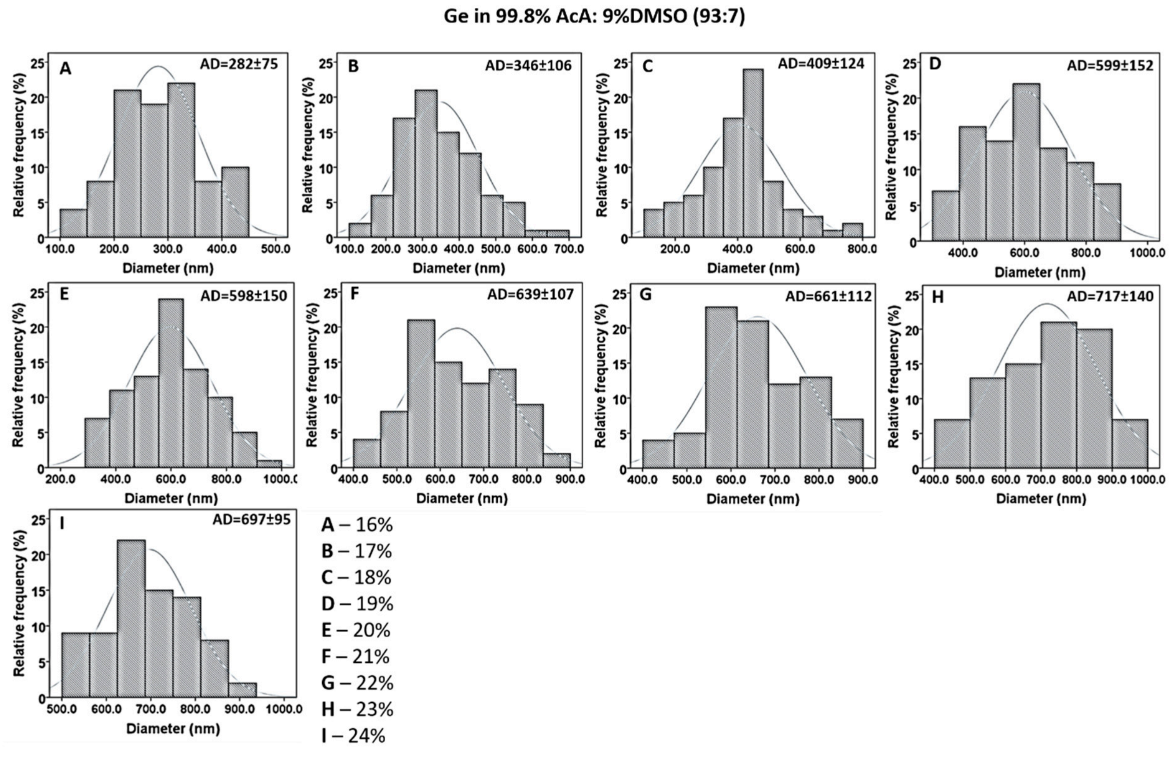

Figure S2. Fiber Diameter Distribution Analysis of Gelatin Electrospun nanofibers for **Series 1: 16-24% Gelatin in 99.8% AcA: 9% DMSO (93:7)**

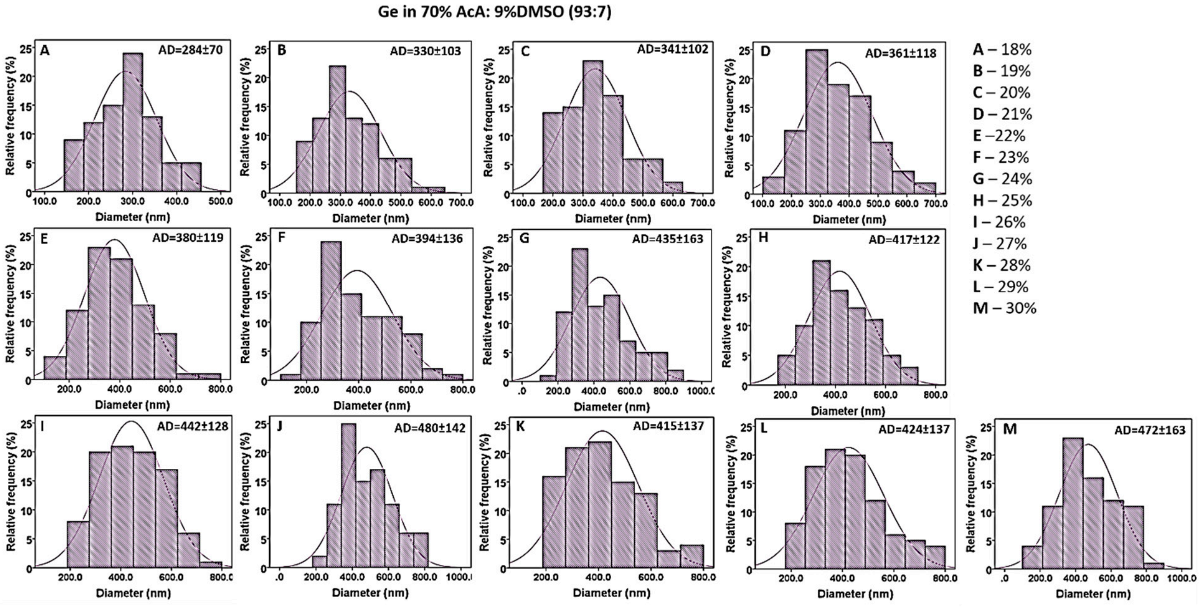

Figure S3. Fiber Diameter Distribution Analysis of Gelatin Electrospun nanofibers for **Series 2: 18-30% Gelatin in 70% AcA: 9% DMSO (93:7)**

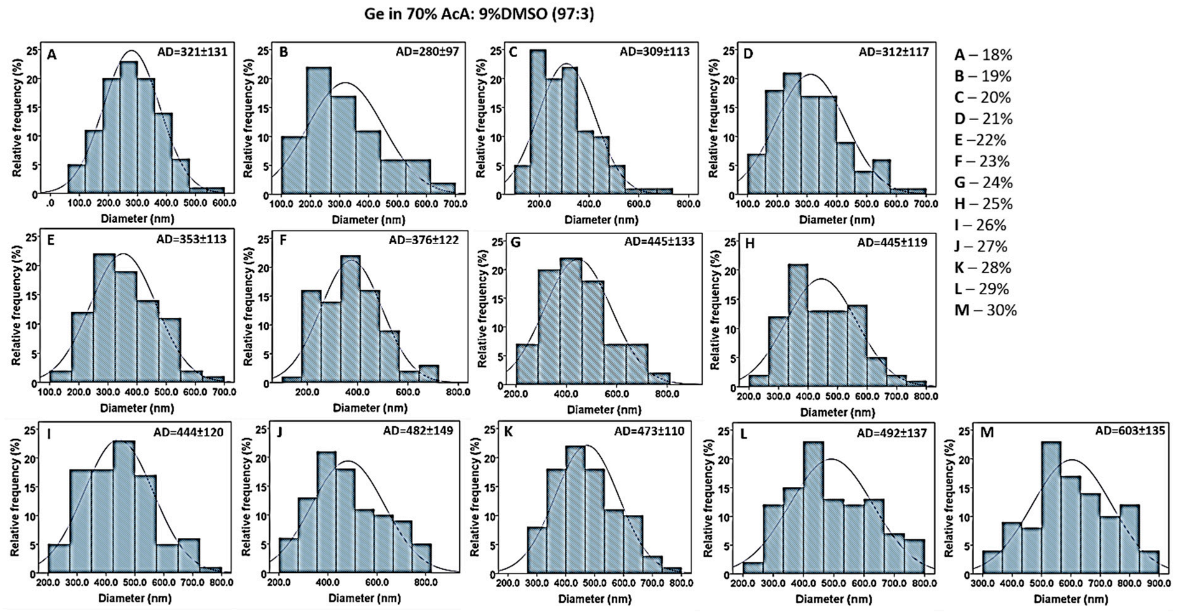

Figure S4. Fiber Diameter Distribution Analysis of Gelatin Electrospun nanofibers for **Series 3: 18-30% Gelatin in 70% AcA: 9% DMSO (97:3)**

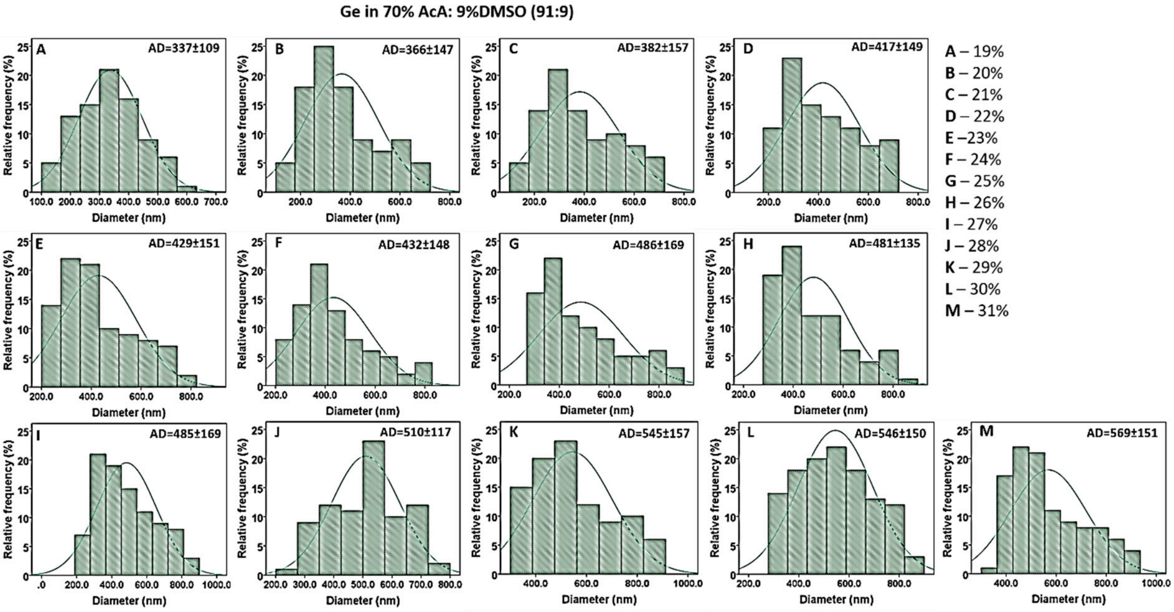

Figure S5. Fiber Diameter Distribution Analysis of Gelatin Electrospun nanofibers for **Series 3: 19-31% Gelatin in 70% AcA: 9% DMSO (91:9)**

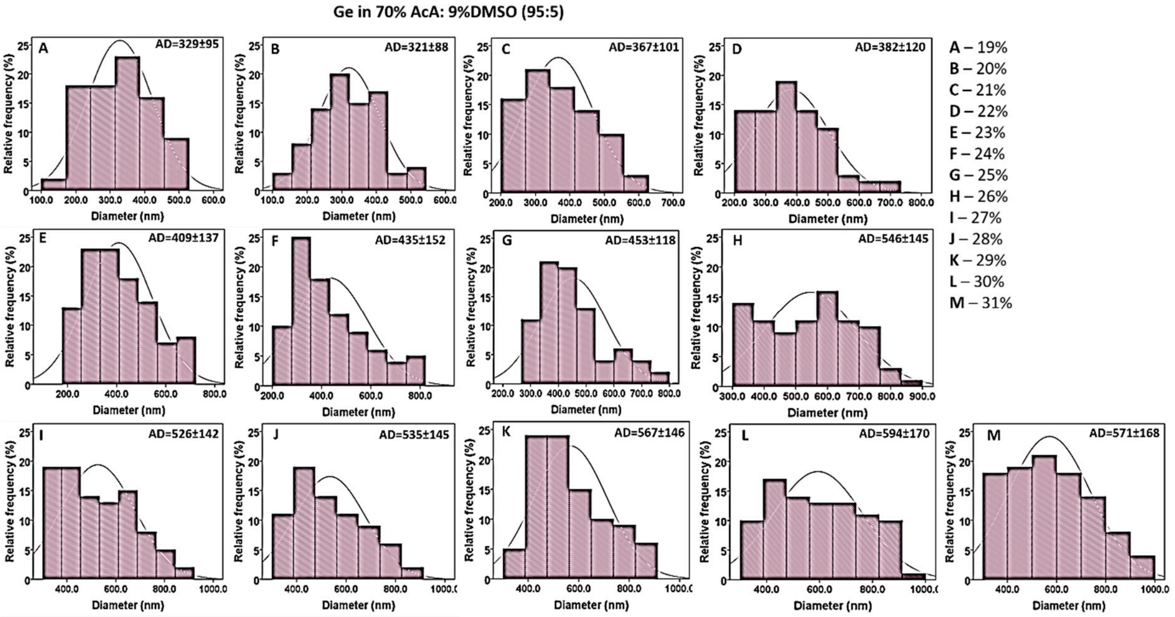

Figure S6. Fiber Diameter Distribution Analysis of Gelatin Electrospun nanofibers for **Series 5: 19-31% Gelatin in 70% AcA: 9% DMSO (95:5)**

FTIR analysis

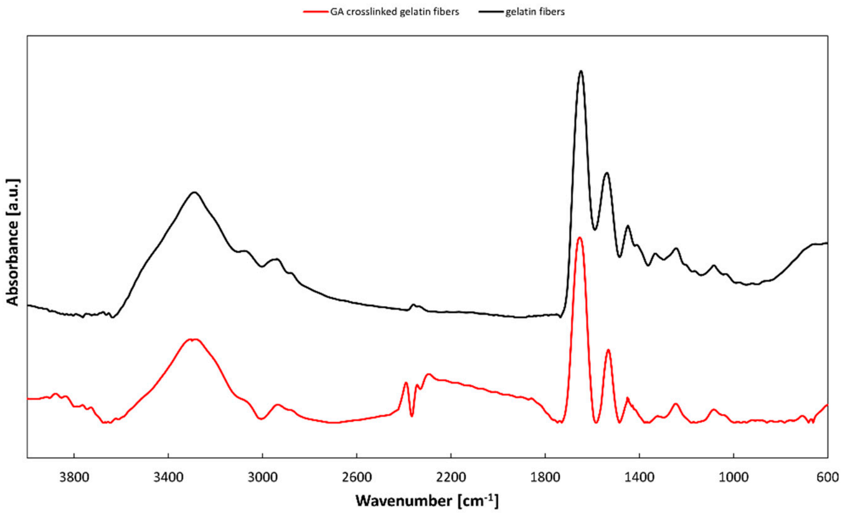

Figure S7. Non-crosslinked gelatin fibers (a) and GA-crosslinked gelatin fibers (b)

Antibacterial properties of drug-free and incorporated antibiotic gelatin scaffolds

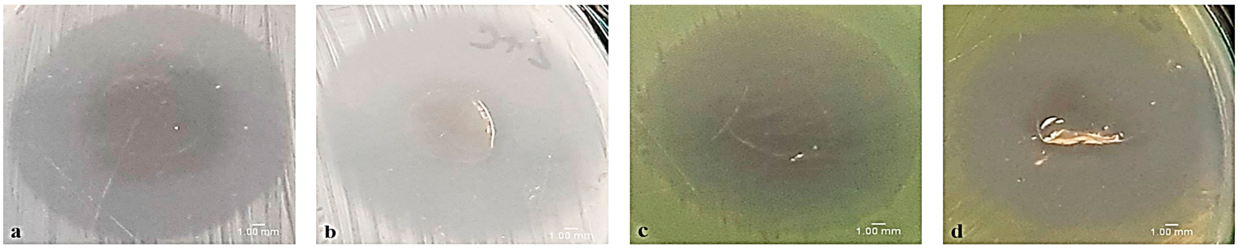

Figure S8. An example of the action of gelatin scaffold: **a)** *Staphylococcus aureus* ATCC 25923 - gelatin/0.1% gentamicin scaffold **b)** *S. aureus* ATCC 25923 - gelatin/0.1% ciprofloxacin scaffold **c)** *Pseudomonas aeruginosa* ATCC 27853 - gelatin/0.1% gentamicin scaffold and **d)** *P. aeruginosa* ATCC 27853 - gelatin/0.1% ciprofloxacin scaffold. The scale bar is 1mm.

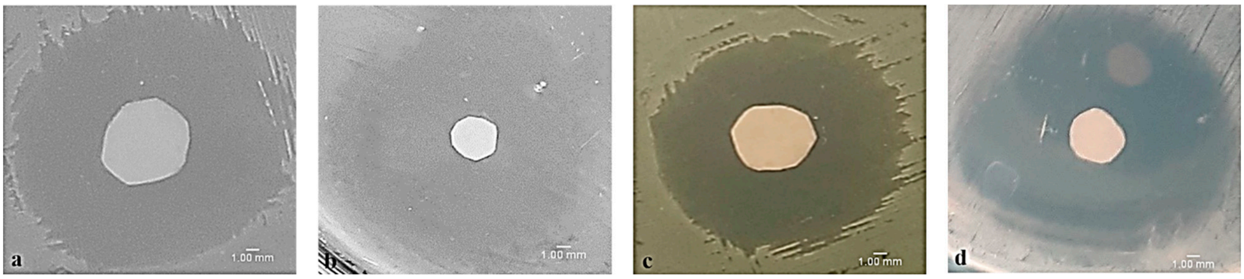

Figure S9. An example of the action of gelatin scaffold: **a)** *Staphylococcus aureus* ATCC 25923 - filter paper/gentamicin 1mg/mL **b)** *S. aureus* ATCC 25923 - filter paper/ciprofloxacin 1mg/mL **c)** *Pseudomonas aeruginosa* ATCC 27853 - filter paper/gentamicin 1mg/mL and **d)** *P. aeruginosa* ATCC 27853 - filter paper/ciprofloxacin 1mg/mL. The scale bar is 1mm.
